# Neural Mechanisms of Cortical Motion Computation Based on a Neuromorphic Sensory System

**DOI:** 10.1371/journal.pone.0142488

**Published:** 2015-11-10

**Authors:** Luma Issa Abdul-Kreem, Heiko Neumann

**Affiliations:** 1 Institute for Neural Information Processing, Ulm University, Ulm, Germany; 2 Control and Systems Engineering Department, University of Technology, Baghdad, Iraq; McGill University, CANADA

## Abstract

The visual cortex analyzes motion information along hierarchically arranged visual areas that interact through bidirectional interconnections. This work suggests a bio-inspired visual model focusing on the interactions of the cortical areas in which a new mechanism of feedforward and feedback processing are introduced. The model uses a neuromorphic vision sensor (silicon retina) that simulates the spike-generation functionality of the biological retina. Our model takes into account two main model visual areas, namely V1 and MT, with different feature selectivities. The initial motion is estimated in model area V1 using spatiotemporal filters to locally detect the direction of motion. Here, we adapt the filtering scheme originally suggested by Adelson and Bergen to make it consistent with the spike representation of the DVS. The responses of area V1 are weighted and pooled by area MT cells which are selective to different velocities, i.e. direction and speed. Such feature selectivity is here derived from compositions of activities in the spatio-temporal domain and integrating over larger space-time regions (receptive fields). In order to account for the bidirectional coupling of cortical areas we match properties of the feature selectivity in both areas for feedback processing. For such linkage we integrate the responses over different speeds along a particular preferred direction. Normalization of activities is carried out over the spatial as well as the feature domains to balance the activities of individual neurons in model areas V1 and MT. Our model was tested using different stimuli that moved in different directions. The results reveal that the error margin between the estimated motion and synthetic ground truth is decreased in area MT comparing with the initial estimation of area V1. In addition, the modulated V1 cell activations shows an enhancement of the initial motion estimation that is steered by feedback signals from MT cells.

## Introduction

Motion perception is a significant source of information for the cortical visual system in which this information is analyzed and processed by a large number of interconnected areas [1]. Each area is characterized by cells of different feature selectivity and varying tuning properties [2]. The neuron responses increase in complexity from the lower level up to the higher cortical area [3, 4]. Motion analysis starts in the primary visual cortex V1 (also known as striate cortex or area 17) [5]. The responses of area V1 are forwarded to subsequent areas, namely middle temporal area (MT), medial superior temporal area (MST) and beyond [6]. Visual areas interact via feedforward and feedback sweep processing (see [1, 7, 8]). The feedforward sweep processes information that are transferred from lower to higher levels via bottom-up connections. These connections are paralleled by top-down connections to project the feedback signals from the higher to the lower level via a reverse counter stream network [1, 9]. Feedback processing tends to act as a modulator to amplify earlier area activations [10].

Although neuroscientists confirm the influence of bidirectional integration among cortical visual areas [1, 11–13], the role of feedback processing is still not fully uncovered [14]. The question is how the higher levels areas can project activations along feedback stream to the lower areas in which the response of each area is characterized by specific properties? In this work, we introduce a model that simulates the visual areas interactions by proposing a new mechanism of feedforward and feedback processing between two main model visual areas with different feature selectivity, V1 (direction selectivity) and MT (velocity selectivity). The initial motion representation generated in model area V1 is weighted and is then subsequently pooled over a local neighborhood defined in the spatio-temporal domain in model MT using cells with larger receptive fields (RFs). Here, a new model of MT motion selective neurons which are tuned to different speeds and directions is introduced.

To enhance the initial motion estimation in area V1 and reduce ambiguity in the estimated motion, top-down feedback signals from area MT are fed back and re-entered in area V1. Along the feedforward path different feature representations of the spatio-temporal input are generated through hierarchically organized filter processes. The specific features extracted are spatio-temporal frequency (in V1) and velocity (direction, speed) at spatial locations (in MT), respectively. The model specifically suggests how MT feature representations generate proper feedback signals that integrate with the spatio-temporal responses in V1. In a nutshell, the re-entry is accomplished through an integration of MT activations over different speed selectivities along a preferred direction. Coarse-grained activations integrated over neighboring direction selectivities emphasis corresponding spatio-temporal directions via modulatory feedback.

To equip the model architecture with realistic input data, we exploit an event-based vision neuromorphic sensor, in which a Dynamic Vision Sensor (DVS) was used instead of a conventional frame-based camera. The DVS sensor simulates the spike-generation functionality of the biological retina [15]. The use of event-based retinas requires an adaptive model that is consistent with the address-event-representation (AER) principle. In our model, we adaptive the spatio-temporal filters that have been suggested by [16] to be consistent with the DVS functionality.

In order to achieve balanced cell activations against the pool of neighboring cells, a normalization process is generated, following [17] and [18]. Here neurons activities are adjusted in the spatial as well as in the feature domains.

Our model can be used as a basis scheme for motion estimation based on sparse events representation. In a further step, the model can be utilized for articulated and biological motions recognition. To evaluate the performance of our model to estimate different motions, we tested the model using different types of stimuli with different translatory and rotational movements.

The paper is organized along the following structure and content. Firstly, we demonstrate the model methodology of V1-MT feedforward interaction. Secondly, we present response normalization in which neurons activities are adjusted in spatial and feature domains. Thirdly, we address the problem of feedback interaction between V1 (direction selectively) and MT (velocity selectively). Fourthly, we present the model results of feedforward sweep processing along V1-MT areas and V1 modulated after feedback processing. Finally, discussion along with a summary of our contributions and future work are introduced.

## Motion Processing Architecture

**Fig 1:**
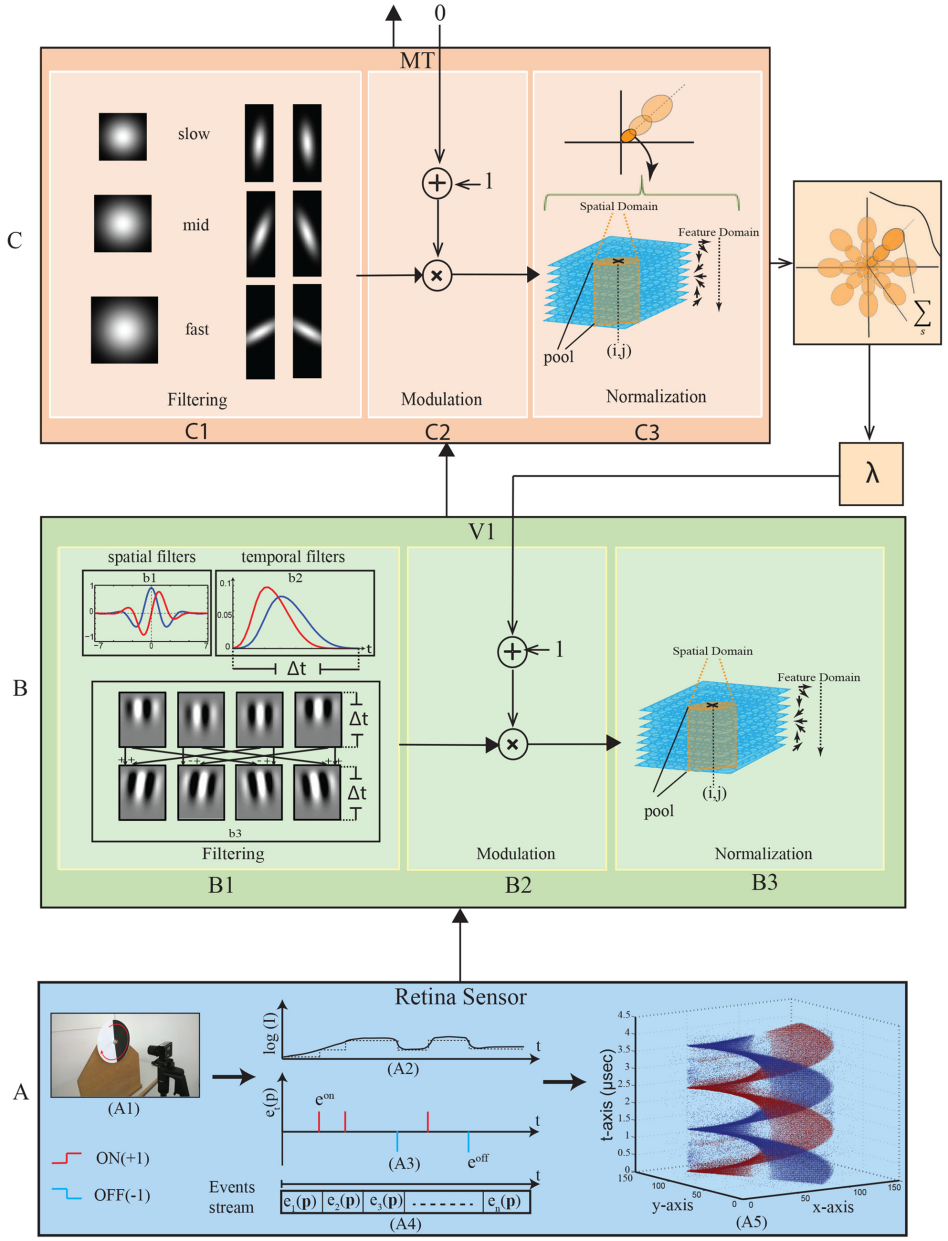
Block diagram of V1-MT feedforward and feedback processing. (A) DVS input. (A1) DVS sensor with half-circular rotational stimulus. (A2) Local changes in intensity (log I) elicit ON or OFF events, depending on the sign of the changes. (A3) *e* ^*on*^ and *e* ^*off*^ identify the event activity (+1) ON and (-1) OFF, respectively. (A4) Event stream which is represented as a sequence of events e at a position **p** and time t. (A5) illustrates the generated events via DVS sensor in 3-dimensional space (x,y,t). (B) The model of area V1 (B1) Spatiotemporal filter construction. (b1) Spatial filters. (b2) Temporal filters. (b3) The first row represents the products of two spatial and two temporal filters; the second row represents the sum and difference of the product filters. (B2) modulation of area V1 based on the feedback of area MT. (B3) normalization mechanism of area V1. (C) The model of area MT. (C1) filtering representation of MT cells. (C2) modulation signal for area MT based on area MST activation which, here, is set to zero. (C3) MT normalizing mechanism.

The architecture of the neural model is inspired by the mammalian visual system in which the input data is evaluated and processed by a hierarchical model of different brain areas. These areas interact through bidirectional feedforward and feedback signal pathways. Motion analysis starts in the primary visual cortex (V1) in which the responses of V1 cells are forwarded to subsequent areas in the model, particularly the middle temporal area (MT) which directly receives V1 projections (see [14, 19]). The action of such re-entrant activation is modulatory in its nature as it cannot by itself generate activity at the reentry target location. In turn, area V1 receives re-entrant input from MT neurons through top-down feedback connections. In addition, the activation in area MT can be modulated through higher level areas such as the medial superior temporal area (MST). Since we do not incorporate a model MST in the current architecture, this modulatory input is left void. Fig 1 shows the hierarchical architecture of V1-MT feedforward and feedback processing in which the model structure of each area is defined by three column stages which are composed of filtering, modulation and normalization. The following subsections detail the overall processing stages of the model.

### Visual input data from the DVS sensor

The functionality of the neuromorphic vision sensor employed here simulates the spike-generation mechanism of the mammalian retina. The sensor is characterized by high temporal resolution, low response latency and large dynamic range visual sensing [20]. The DVS exploits the Address-Event-Representation (AER) principle in which each pixel of the vision sensor responds independently with a high temporal resolution (1 *μs*) and can be influenced by a small spectral neighborhood to a accomplished center-surround interaction like in the retina or LGN [20]. The neuromorphic sensing emulates biological retinal sensing and spike-based neuronal processing in which spike events are triggered when an intensity change is detected [21]. The DVS uses the polarity format for event representation, namely ON (+1) for positive intensity (log I) change and OFF (-1) for negative intensity (log I) change of above threshold difference. Zero output, however, is produced when no change in intensity (log I) is detected which in turn discards any redundant information in comparison to frame-based cameras. Fig 1A shows an example of the DVS spike events in response to rotational stimulus. The DVS sensor generates spikes, or events, where each recorded event is indexed using the 2D spatial location (x,y), the polarity of the luminance change (ON or OFF), and the time-stamp of the event. We exploit the neuromorphic vision sensor to pursue the bio-inspired model for motion estimation which is a continuation of our own previous work reported in [22] and builds upon recent theoretical work reported in [23]. Since a single event in spatio-temporal domain gives rise an ambiguity for motion estimation using spatiotemporal filtering, pixel activity ON (+1) and OFF (-1) is accumulated during a temporal window. The accumulation of the ON/OFF events is described by *e*(**p**, *t*) = *e* ^*on*^(**p**, *t*) + *e* ^*off*^(**p**, *t*) where *e* ^*on*^(**p**, *t*) and *e* ^*off*^(**p**, *t*) represent ON and OFF events respectively, occurring at position **p** = (*x*, *y*) and time *t*. The interval length of the temporal window can be parametrized in accordance with the extent of the temporal impulse responses of the filter functions employed.

### Motion estimation-feedforward processing

In our model, motion is estimated initially by cells in area V1 which are selective to movement direction. Due to the small RFs of V1 neurons local motion can only be detected along direction orthogonal to extended contrasts (i.e., aperture problem). The local estimated motion is, then, weighted and integrated in the next visual area (MT). Here, cells are modeled using new mechanism which are selective to different directions and speeds. In the following subsections the proposed models of the two areas are described.

#### Model mechanism for area V1

We slightly modified the mechanism of the filtering stage model that was suggested in [22] in which the motion energy model of [16] was adapted to be compatible with the AER principle. Our model employs spatiotemporal filters, which are analogous to the RFs of cells in the primary visual cortex. These filters can be deconstructed into two 2D kernels in the space domain and two 1D kernels in the temporal domain. The temporal filters use two different integration windows (fast and slow), while the spatial filter uses two different phases (even and odd). Fig 1B1(b1) shows the two spatial filters (even and odd), in which 2D Gabor functions, Eqs (1) and (2), are used to implement even and odd symmetric filters, respectively

$$\begin{array}{c} F_{even}\left(x,y,\theta_{k},f_{s}\right)=\frac{1}{2\pi \sigma_{s}^{2}}\cdot exp\left(-\frac{{\overset{˘}{x}}^{2}+{\overset{˘}{y}}^{2}}{2\sigma_{s}^{2}}\right)\cdot cos\left(2\pi f_{s}\overset{˘}{x}\right), \end{array}$$ (1)

$$\begin{array}{c} F_{odd}\left(x,y,\theta_{k},f_{s}\right)=\frac{1}{2\pi \sigma_{s}^{2}}\cdot exp\left(-\frac{{\overset{˘}{x}}^{2}+{\overset{˘}{y}}^{2}}{2\sigma_{s}^{2}}\right)\cdot sin\left(2\pi f_{s}\overset{˘}{x}\right), \end{array}$$ (2)

where $\left(\begin{array}{c} \overset{ˇ}{x}\\ \overset{ˇ}{y} \end{array})=(\begin{array}{cc} cos\theta_{k} & -sin\theta_{k}\\ sin\theta_{k} & cos\theta_{k} \end{array})\cdot (\begin{array}{c} x\\ y \end{array}\right)$, *θ*_*k*_ is the spatial filter orientation with *N* different orientations where *k* = {1, 2, 3 … *N*}, *σ*_*s*_ is the standard deviation of the spatial filters, and *f*_*s*_ represents the spatial frequency tuning.

In [16], the authors used temporal gamma functions (*f*(*t*) = (*kt*)^2^ ⋅ *exp*(−*kt* ^2^) ⋅ [1/*n*! − (*kt*)^2^/(*n* + 2)!]) of different durations (n = 3, n = 5) in order to achieve temporal smoothing and differentiation. Since event-based sensor responses already encode temporal luminance changes, i.e. temporal derivatives of the input signals, we employ a convolution process utilizing smoothing temporal filters (Eq (3)) which integrate the input stream of events.

$$\begin{array}{c} f\left(t\right)=\int {\left(kt\right)}^{2}\cdot exp\left(-kt^{2}\right)\cdot \left[1/n!-{\left(kt\right)}^{2}/\left(n+2\right)!\right]. \end{array}$$ (3)

**Fig 2:**
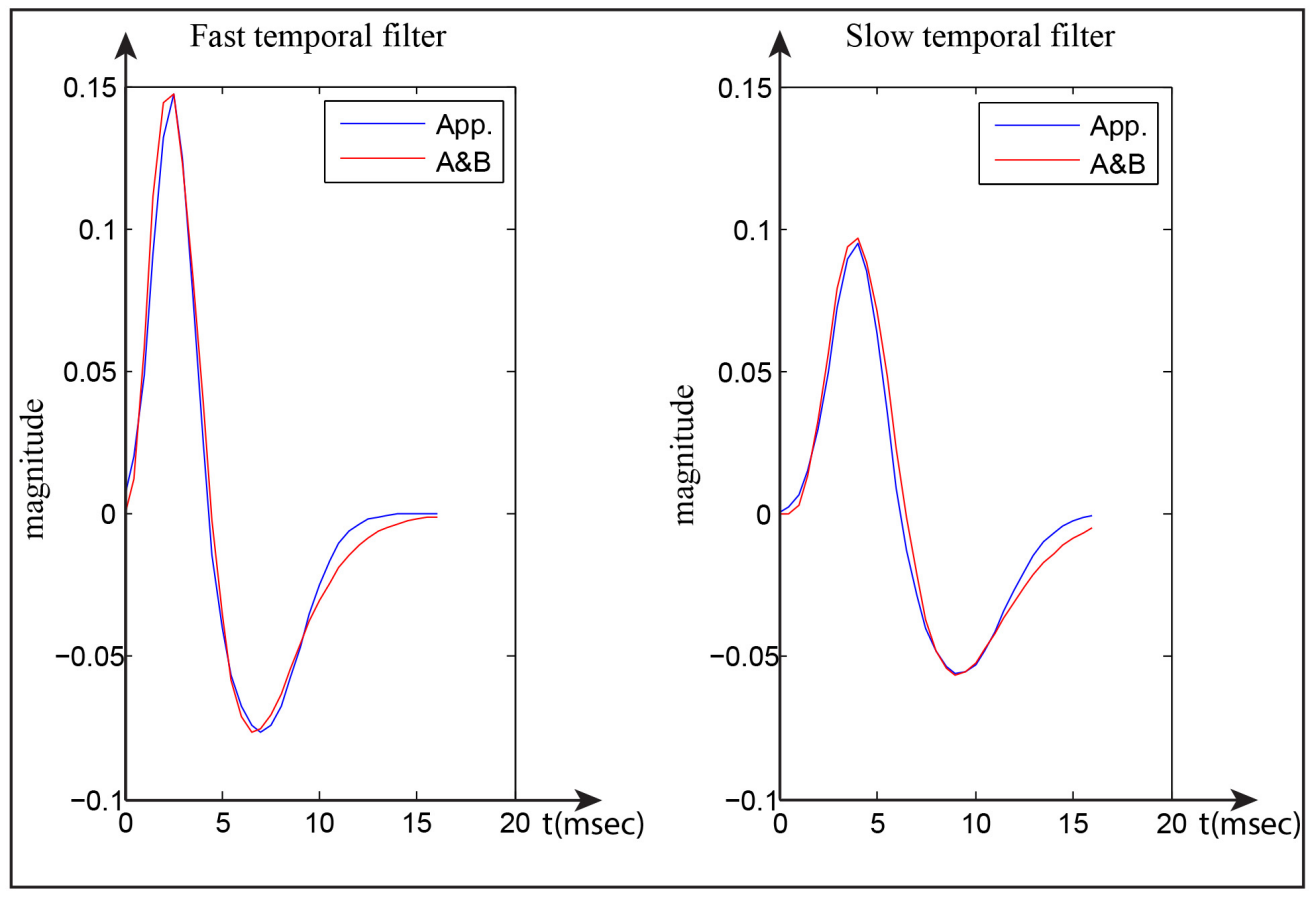
Fast and slow temporal filters. Red lines show Adelson-Bergen filters and blue lines show the approximated response derived by two shifted Gaussian envelopes as filters.

This allows us to obtain scaled versions of temporally smoothed derivatives of the input luminance function as input representation for motion estimation. To simplify the mathematical description of the temporal filters in Eq (3), we suggest to combine two Gaussian functions (Λ) having different standard deviations(*σ*) and mean values (*μ*). This combination is scaled by a scale factor *c* to closely resemble the shape of the Adelson-Bergen temporal filters as given in Eq (4)

$$\begin{array}{c} S_{slow,fast}\left(t\right)=[\Lambda_{\sigma_{1},\mu_{1}}\left(t\right)-\Lambda_{\sigma_{2},\mu_{2}}\left(t\right)]/c, \end{array}$$ (4)

with $\Lambda_{\sigma_{*},\mu_{*}}\left(t\right)=1/\left(\sigma_{*}\sqrt{2\pi }\right)\;\cdot exp\left(-{\left(t-\mu_{*}\right)}^{2}/\left(2\sigma_{*}^{2}\right)\right)$, where μ_*_ and σ_*_ represent the mean and the width of temporal window extent of the Gaussian function, respectively. Fig 2 shows the temporal filters (fast and slow) of Adelson-Bergen and the approximation responses of the Gaussian functions combination. We used *σ*_1_ = 1, *μ*_1_ = 2.5, *σ*_2_ = 2, *μ*_2_ = 7, *c* = 2.6 to generate the fast temporal filter and *σ*_1_ = 1.3, *μ*_1_ = 4, *σ*_2_ = 2.3, *μ*_2_ = 9.2, *c* = 3.1 to generate the slow temporal filter. Subsequently, the smoothing temporal filters *T*_*slow*, *fast*_(*t*) are calculated based on the integral ∫*S*_*slow*, *fast*_(*t*)*dt*, Eq (4), and then the summation of each filter is scaled to 1 to prevent any biases in calculating responses. The smoothing temporal filters are thus given by

$$\begin{array}{c} T_{slow,fast}\left(t\right)=\frac{1}{\int_{0}^{\infty }\left(G_{1}-G_{2}\right)dt}\cdot \left(G_{1}-G_{2}\right)\left(t\right), \end{array}$$ (5)

$$\begin{array}{c} G_{1}=[\frac{1}{2}(1+erf(\frac{\left(t-\mu_{1}\right)}{\sigma_{1}\sqrt{2}})]/c, \end{array}$$ (6)

$$\begin{array}{c} G_{2}=[\frac{1}{2}(1+erf(\frac{\left(t-\mu_{2}\right)}{\sigma_{2}\sqrt{2}})]/c, \end{array}$$ (7)

where *T*_*slow*, *fast*_(*t*) defines the fast and slow temporal filters, *G*_1_ and *G*_2_ represent the integral of Gaussian functions ∫Λ_*σ*_1_, *μ*_1__(*t*)*dt* and ∫Λ_*σ*_2_, *μ*_2__(*t*)*dt*, respectively. Fig 1B1(b2) shows the fast and slow temporal filters.

The spatiotemporal separable filters were calculated according to the scheme proposed in [16] in which the products of two spatial and two temporal filters are shown in the first row of Fig 1B1(b3). These filters are combined in a linear fashion in order to obtain the oriented selectivity in the spatio-temporal domain, as shown in the second row of Fig 1B1(b3). We get the filter functions below

$$\begin{array}{c} F_{a}^{v1}\left(x,y,\theta_{k},f_{s},t\right)=F_{even}\left(x,y,\theta_{k},f_{s}\right)\cdot T_{slow}\left(t\right)+F_{odd}\left(x,y,\theta_{k},f_{s}\right)\cdot T_{fast}\left(t\right), \end{array}$$ (8)

$$\begin{array}{c} F_{b}^{v1}\left(x,y,\theta_{k},f_{s},t\right)=F_{even}\left(x,y,\theta_{k},f_{s}\right)\cdot T_{fast}\left(t\right)-F_{odd}\left(x,y,\theta_{k},f_{s}\right)\cdot T_{slow}\left(t\right). \end{array}$$ (9)

The filtering response of area V1 for a stream of events input *e*(*x*, *y*, *t*) can be calculated by the gradual activation response function ${\dot{r}}^{v1}=-r^{v1}+\left({\left[F_{a}^{v1}*e\right]}^{2}+{\left[F_{b}^{v1}*e\right]}^{2}\right)$ which has been solved at equilibrium state

$$\begin{array}{c} r_{\theta }^{v1}=\left({\left[F_{a}^{v1}*e\right]}^{2}+{\left[F_{b}^{v1}*e\right]}^{2}\right). \end{array}$$ (10)

The symbol ‘*’ indicates the convolution operator, *θ* indicates motion directions (left vs. right relative to the orientation axis). For better readability we omitted the local spatial coordinates and feature selectivities.

#### Model mechanism for area MT

**Fig 3:**
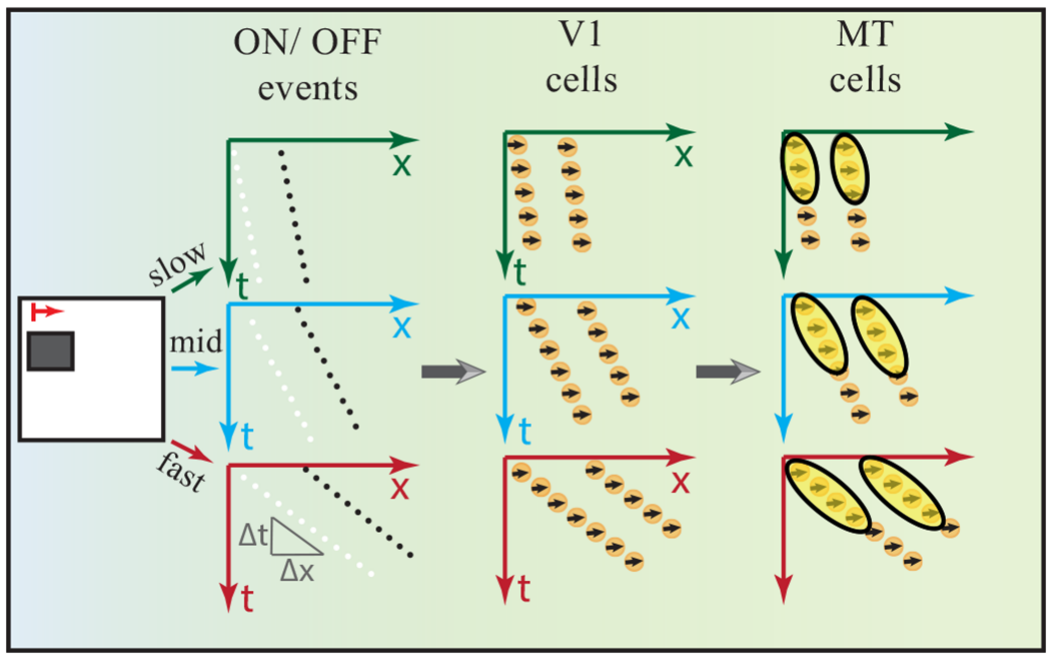
speed representation in spatiotemporal (x,t) domain. A rectangular object is moving from left to right at different speeds. In the first column, streams of ON/OFF events are generated in the spatiotemporal domain (x,t) for different speeds (slow, mid, fast). The sequences of oriented in the space-time domain corresponding to the speed of the motion. In the second column, sketch of the activated V1 cells in the 2D spatiotemporal domain driven by a slow, mid and fast sweep of input stimulus. The third column shows how cells in area MT encode the speed of the motion via integrate spots of V1 activations in spatiotemporal domain.

**Fig 4:**
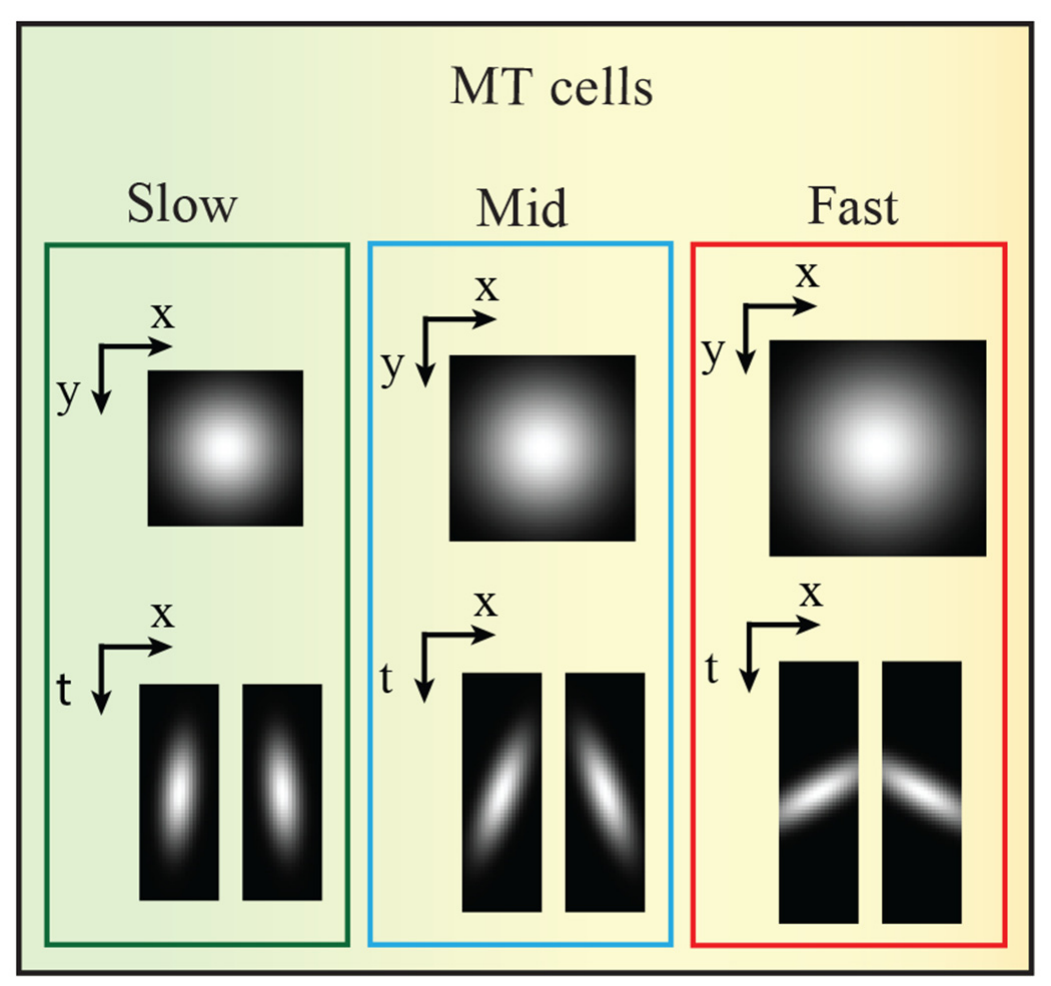
The model of area MT. The RFs of cells in area MT are modeled using Gaussian functions with circular and elongated shapes.

In area MT, again, the structural model is defined by three stages, namely filtering, modulation, and normalization, as shown in Fig 1C. In the filtering stage, the incoming visual responses from area V1 are weighted and pooled with larger RFs. Here, we propose a neural model for area MT where neurons are selective to different velocities. In essence, we modeled the neurons in area MT by taking into account the following features:

1. Speed and direction selectivity of MT cells encoded via V1 cells: Cells in area V1 which are driven by a sweep of input stimulation are distributed over a larger spatial field of locations. Due to their small RFs such cells respond spatio-temporally rather coarsely, but can distinguish directions (left vs. right orthogonal to the contrast orientation). In area MT, cells have larger RFs that integrate such spots of V1 activations for a given speed-range. Thus, speed representation in the MT neurons can be encoded via the V1 cells population with respect to the temporal axis. Fig 3 shows rectangular object moving from left to right at different speeds (slow, mid, fast). The translation of the object corresponds to an oblique stream of ON/OFF events in the spatiotemporal (x,t) domain (we omitted the y-component to keep illustrations simple). The slope of the distributed stream of events reflects the speed of the motion. Cells in area V1 are activated according to these distributions of events and thus generate a population of activated cells that are oriented in spatio-temporal domain. The responses of V1 cells are integrated in the space-time domain via MT cells with larger RFs. This kind of integration equips MT cells with contextual information for a particular speed-range.
2. Organization and geometry of MT cells: The RFs geometry of MT neurons has been investigated by [24], where the authors identified two types of MT RFs profiles: classical receptive field CRF and center-surround cells. CRF responds best to wide field motion, while center-surround cells are sensitive to motion contrasts, which can be described by center-surround interaction. We focus on CRF cells in our model in which weighting functions with different sizes are utilized. These cells are organized in the spatial-temporal domain (x,y,t). We use Gaussian fall-off functions to integrate the spatial responses of V1 cells over larger RFs (V1:MT 1:3, 1:3.5 and 1:4). In addition, elongated weighting filters are used to integrate the responses of V1 cells population with respect to the temporal axis. The integration process of V1 responses over time axis encodes the speed representation in area MT. The elongated weighting filters are tuned to different speeds (fast, mid, and slow) by orienting them in the spatiotemporal domain in which fast motion tuning filters are more oriented with respect to the temporal axis. Following the study of [25], we increased the size of the RF profiles with increasing speed selectivity. Fig 4 shows the model of MT cells. The responses of such cells are calculated by
  $$\begin{array}{c} r_{\theta ,s_{i}}^{MT}\left(x,y,t\right)=\sum_{\bar{\phi }}{\left\{r_{\theta }^{v1}*\Lambda_{\sigma_{i}}*\Psi_{{\bar{\phi }}_{i}}\right\}}_{x,y,t}, \end{array}$$ (11)
  $$\begin{array}{c} \Lambda_{\sigma_{i}}=\frac{1}{2\pi \sigma_{i}^{2}}\cdot exp(-\frac{x^{2}+y^{2}}{2\sigma_{i}^{2}}), \end{array}$$ (12)
  $$\begin{array}{c} \Psi_{{\bar{\phi }}_{i}}=\frac{1}{2\pi \sigma_{x}\sigma_{t}}\cdot exp(-\frac{{\overset{ˇ}{x}}^{2}}{2\sigma_{x}^{2}}+\frac{{\overset{ˇ}{t}}^{2}}{2\sigma_{t}^{2}}), \end{array}$$ (13)
  where $\left(\begin{array}{c} \overset{ˇ}{x}\\ \overset{ˇ}{t} \end{array})=(\begin{array}{cc} cos{\bar{\phi }}_{i} & -sin{\bar{\phi }}_{i}\\ sin{\bar{\phi }}_{i} & cos{\bar{\phi }}_{i} \end{array})\cdot (\begin{array}{c} x\\ t \end{array}\right)$, the symbol ‘*’ indicates the convolution operator, Λ_*σ*_*i*__ denotes the fall-off Gaussian weighting functions in which *σ*_*i*_ is the spatial extent, $\Psi_{{\bar{\phi }}_{i}}$ represents the elongated Gaussian functions where ${\bar{\phi }}_{i}$ is the orientation of the Gaussian function (left vs. right relative to the orientation axis *ϕ*_*i*_, −*ϕ*_*i*_), $r_{\theta }^{v1}$ is the response of area V1 in which *θ* denotes motion direction selectivity, the index *s*_*i*_ represents speed selectivity *s*_*i*_ = {*fast*, *mid*, *slow*} that corresponds to the spatial extent *σ*_*i*_ and the orientation ${\bar{\phi }}_{i}$.
3. Temporal integration: We incorporated a temporal trace rule suggested by [26] in our model in order to hold an amount of past neural activations in which the weight of the present activities is higher than the past activities. The extent of such temporal windows takes into account the temporal impulse responses of MT cells reported in, e.g., [27]. Such a rule has also been adopted in several studies (e.g., [28, 29]).
  $$\begin{array}{c} {\dot{r}}_{s_{i},\theta }^{MT_{out}}(t)=-\delta r_{s_{i},\theta }^{MT_{out}}\left(t\right)+r_{s_{i},\theta }^{MT_{in}}\left(t\right), \end{array}$$ (14)
  where *δ* (0 < *δ* < 1) represents the strength of the temporal integration and ${\dot{r}}_{s_{i},\theta }^{MT_{out}}(t)$ denotes the calculated average activity of MT cells, $r_{s_{i},\theta }^{MT_{out}}\left(t\right)$ and $r_{s_{i},\theta }^{MT_{in}}\left(t\right)$ represent the input and output activities of the MT cells.

## Response Normalization

Experimental investigation have shown that the responses of cells in visual cortical areas show significant nonlinearities depending on spatio-temporal activity distribution in the cell activation in the space-feature domain surrounding a target cell [30, 31]. Such response nonlinearities have been demonstrated in the LGN, early visual cortex (area V1), and beyond. In many studies, it has been proposed that responses are calculated via the normalization of the target cell response based on pooling activities in a neighborhood (see [18, 32]). Model neuronal activation is described by gradual changes of the membrane potential which is here considered as a population state variable, as described by [33]

$$\begin{array}{c} \tau \frac{dv(t)}{dt}=-A\cdot v(t)+(B-C\cdot v(t))\cdot net_{ex}-(D+E\cdot v(t))\cdot net_{in}, \end{array}$$ (15)

where *A* represents the passive leakage, *B* and *D* are parameters denoting the saturation potentials (relative to *C* and *E*, respectively), and *net*_*ex*_ and *net*_*in*_ denote generic excitatory and inhibitory inputs to the target cell with the state, or membrane potential. Here, the net inputs are defined by the responses encoding motion from the filtering stage.

A firing-rate function generates positive output activations from the current state level, or membrane potential. Here, we employ a simple rectification to yield *r*(*t*) = *max*(0, *v*(*t*)). We set the parameters in Eq (15) such that the output range for the membrane potential is bounded to the range [0, *B*], setting B = 1 and D = 0 and C = E = 1.

In order to achieve balanced activities of individual cells against neighborhood activities, we employ a normalization mechanism in which neuron activities are adjusted in the spatial and feature domains. We solved Eq (15) at equilibrium to drive the steady-state response $\frac{dv(t)}{dt}=0$, using the parametrization for constants A to E as defined above

$$\begin{array}{c} v_{\infty }=\frac{net_{ex}}{A+net_{ex}+net_{in}}. \end{array}$$ (16)

As outlined in Fig 1 the activities of areas V1 and MT are normalized in the third stage B3 and C3, of each individual processing cascade, respectively. The normalization of the V1 motion selective responses take the spatial and the feature domain into account. Here, the features considered are orientations and directions. The model responses *r* ^*v*1^ are normalized in the spatial domain using a Gaussian weighting function defined over the spatial domain. The motion selective responses are defined in orthogonal direction space relative to the local contrast orientation *θ* of the spatial filter kernels used. We take the direction feature space into account as well by summing activity over all directions and then scaling this sum by *N*. In all, we can denote the overall pool activation by

$$\begin{array}{c} r^{v1pool}\left(x,y\right)=\frac{1}{N}\sum_{\theta }{\left\{r_{\theta }^{v1}*\Lambda_{\sigma_{nor}}\right\}}_{x,y}, \end{array}$$ (17)

with *θ* denoting the motion directions, ‘*’ denotes the (spatial) convolution operator, N is the number of contrast filter orientations and Λ is the spatial weighting function of the pooling operation. The latter is a Gaussian function (as defined in Eq (12)) which is parametrized by the parameter *σ*_*nor*_ to denote the width of the spatial extent. Finally, the resulting normalized responses in the spatial and feature domains is calculated by

$$\begin{array}{c} r_{\theta }^{v1nor}\left(x,y\right)=\frac{r_{\theta }^{v1}\left(x,y\right)}{A+r_{\theta }^{v1}\left(x,y\right)+r^{v1pool}\left(x,y\right)}. \end{array}$$ (18)

Like in Eq (16) constant parameter *A* refers to the passive decay of the corresponding dynamic mechanism and avoids zero division.

Similarly, the activity of individual neurons in areas MT are balanced in the spatial domain as well as in the feature domain. Here, features are defined in the velocity space (both directions and speed). In the spatial domain, again, we used a fall-off function Λ_*σ*_*nor*__ to weight the activity in the spatial neighborhood of the target cell according to the width of the spatial extent *σ*_*nor*_. While in the feature domain, the motion selective responses of MT neurons are defined in velocity space (direction and speed). We adjust cell activations against the pool of neighboring cells in the direction domain at each particular speed. This is accomplished by averaging activities of each cell over all directions {*θ*_*k*_}_*k* = 1···*N*_ of each speed *s*_*i*_. The pooling activities of individual neuron over spatial and feature domain are defined by

$$\begin{array}{c} r_{s_{i}}^{MTpool}\left(x,y\right)=\frac{1}{N}\sum_{\theta }{\left\{r_{\theta ,s_{i}}^{MT}*\Lambda_{\sigma_{nor}}\right\}}_{x,y}, \end{array}$$ (19)

where the index *s*_*i*_ represents the speeds, *i* = {1···3} corresponds to (slow, mid and fast). The resulting normalized response of neurons in area MT in the spatial and feature domain is finally calculated by

$$\begin{array}{c} r_{\theta ,s_{i}}^{MTnor}\left(x,y\right)=\frac{r_{\theta ,s_{i}}^{MT}\left(x,y\right)}{A+r_{\theta ,s_{i}}^{MT}\left(x,y\right)+r_{s_{i}}^{MTpool}\left(x,y\right)}. \end{array}$$ (20)

Again constant parameter *A* refers to the passive decay of the corresponding dynamic mechanism and avoids zero division.

## Feedback from Area MT to V1

Along the feedforward processing sweep the feature representations in area V1 and MT, respectively, are different. Cells in area V1 are direction-selective while cells in area MT are velocity-selective (direction and speed). In other words, the bottom-up signals (V1→MT) predominantly drive from direction features (and do not distinguish between different speeds), while the top-down signals (MT→V1) drive from both direction and speed features. The question here is how can the higher area signals interact with the lower area where both are fed by different feature domains? We propose integrating different speed responses along a particular preferred direction. This integration transforms the feature responses of the MT cells from direction and speed domains to the direction domain. Here, the higher area MT signals can be re-entered at the lower area V1, in which the properties of both areas are matched. In order to keep the smoothness of the top-down signals in the feature-space, we weighted the activities in MT over the neighborhood directions using a Gaussian function. Area V1 cells receive feedback signals from the higher area MT such that the feedforward activities can be modified via the modulatory influence of feedback signals. The top-down signals alone, however, cannot provoke any activities when feedforward signals are absent. We utilized the feedback modulatory mechanism suggested by [34, 35], which in abstract terms is denoted by,

$$\begin{array}{c} r_{p,feat}^{out}\propto r_{p,feat}^{FF}\cdot \left(1+\lambda \cdot r_{p,feat}^{FB}\right). \end{array}$$ (21)

In this scheme *FF* denotes to the feedforward signal stream and *FB* refers to the feedback stream. The indices **p** and *feat* represent the spatial position **p** = (*x*, *y*) and the considered feature, and *λ* denotes a constant amplification factor. Based on this, the modulated V1 responses in our model are calculated by

$$\begin{array}{c} r_{x,y,\theta }^{v1_{mod}}=r_{x,y,\theta }^{V1}\cdot \left(1+\lambda \cdot \sum_{s_{i}}r_{\theta ,s_{i}}^{MT_{nor}}\cdot \Lambda_{\sigma_{m}}\right), \end{array}$$ (22)

where Λ_*σ*_*m*__ is Gaussian weighting function of the smoothing operation over direction *θ* in which *σ*_*m*_ denotes the width of the smoothing extent, *s*_*i*_ refers to discrete speed ranges, indexed by *i* = {1···3} and *λ* defines the strength of the feedback projection from area MT. In the case of a pure feedforward processing sweep, the modulator feedback signals are switched off by setting *λ* = 0. As a consequence, the filtering response signals in model area V1 are forwarded to the third column stage in which the responses of V1 cells are normalized in the spatial and feature domains.

## Results

To demonstrate the potential of our model, we used synthetic ground truth data along with a set of different stimuli with translator and rotational motions. The motions of the stimuli were recorded using the dynamic visual sensor (DVS 128 sensor) that introduced by [20]. In our model, the size of the spatial filters in area V1 has been defined as a function of the spatial frequency [36] and [37] such that $\sigma_{s}=\frac{0.5622}{f_{s}}$. This parametrization is taken in accordance to suggestion in [38]. We have used a spatial frequency of *f*_*s*_ = 0.25 in which the size of the spatial kernel in area V1 is (15 × 15). The estimated motion of our result is based on 8 directions *θ* = {0°,45°,90°,135°,180°,225°,270°,315°}. In the filtering stage of area MT the sizes of the fall-off kernels in the spatial (x,y) domain are (45 × 45), (49 × 49) and (53 × 53) pixels for the slow, mid and fast motion, respectively. These sizes keep the integration process in area MT with larger RFs (V1:MT 1:3, 1:3.5 and 1:4). The sizes of the elongated Gaussian filters in spatiotemporal (x,t) domain are (21 × 57), (21 × 63) and (21 × 69) which are oriented by (240°(left), 300°(right)), (280°(left), 340°(right)) and (175°(left), 355°(right)) for slow, mid and fast motion respectively. In order to hold an amount of past neural activation, *δ* has been set to 0.5. In the normalization stage of areas V1 and MT, we set the standard deviation *σ*_*nor*_ of the spatial weighing function to 15 which exceeds the size of the RFs of both areas. Here, the passive decay parameter is set to a small value (*A* = 0.01) to avoid zero division. In the feedback processing stream we set *λ* to 0.8 and *σ*_*m*_ to 2. The following subsections introduce our experimental results to probe the speed sensitivity in area MT. In addition, motion representation in areas V1 and MT via feedforward processing stream will be demonstrated along with the motion representation of the modulated V1 via feedback processing stream.

### Speed selectivity in areas V1 and MT

**Fig 5:**
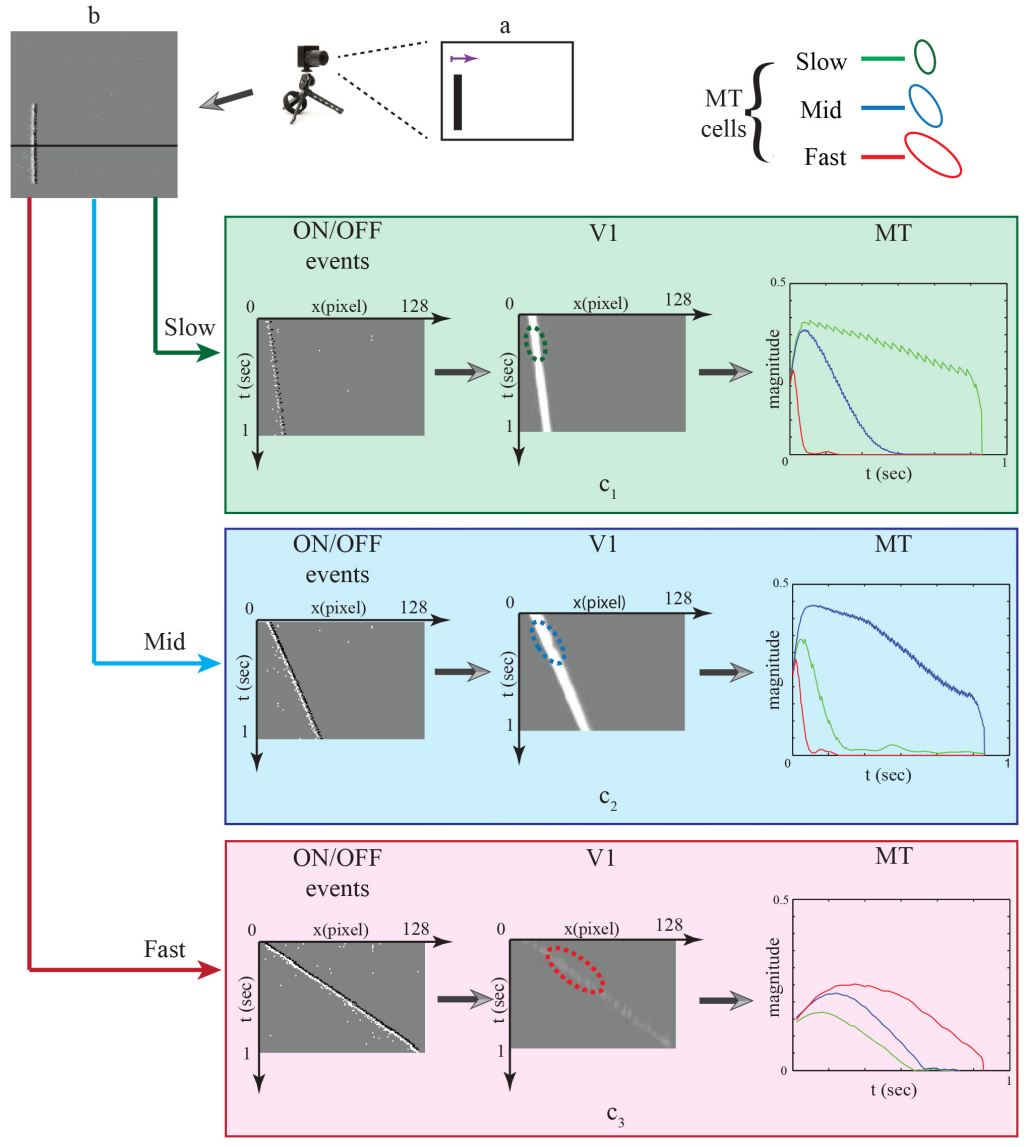
Bar movement with three different speeds. The bar was moved to the right with three speeds (slow 7 *cm*/*sec*, medium 17 *cm*/*sec* and fast 23 *cm*/*sec*). (a) Shows the input stimulus. (b) The representation of the ON/OFF spike responses that are generated via the DVS 128 sensor. (*c*_1_) The ON/OFF events in the spatial-temporal domain of the bar movement to the rightward with slow motion. (*c*_2_) and (*c*_3_) show the spatial-temporal domain of the bar movement to the rightward with mid and fast motions, respectively. The responses of slow, mid and fast selective MT cells are depicted by solid-green, solid-blue and solid-red lines, respectively. The slow, mid and fast selective cells are depicted over V1 responses as dashed-green, dashed-blue and dashed-red, respectively.

**Fig 6:**
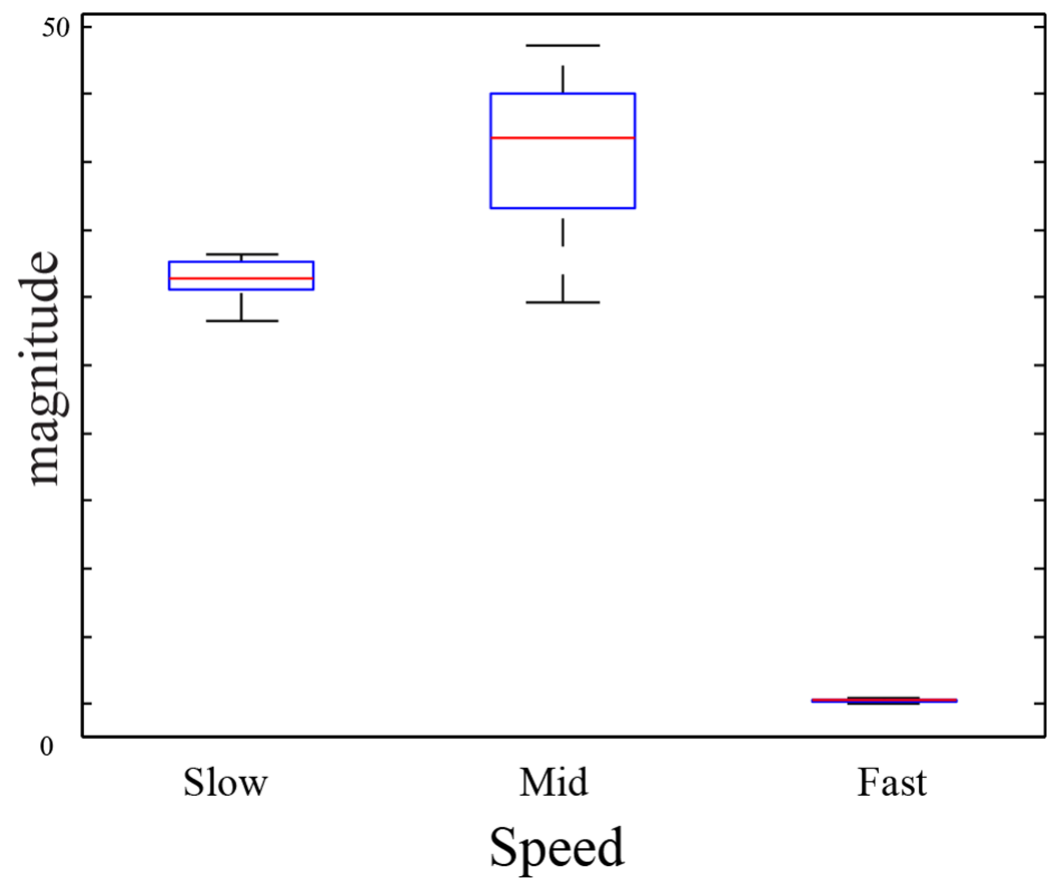
The distribution of V1 responses. The first box shows the distribution of the responses in area V1 which are driven by slow bar motion (slow 7 *cm*/*sec*). The second and third boxes show the output distribution of area V1 cells (filters) in response to mid and fast bar motion (17 *cm*/*sec* and 23 *cm*/*sec*), respectively.

**Fig 7:**
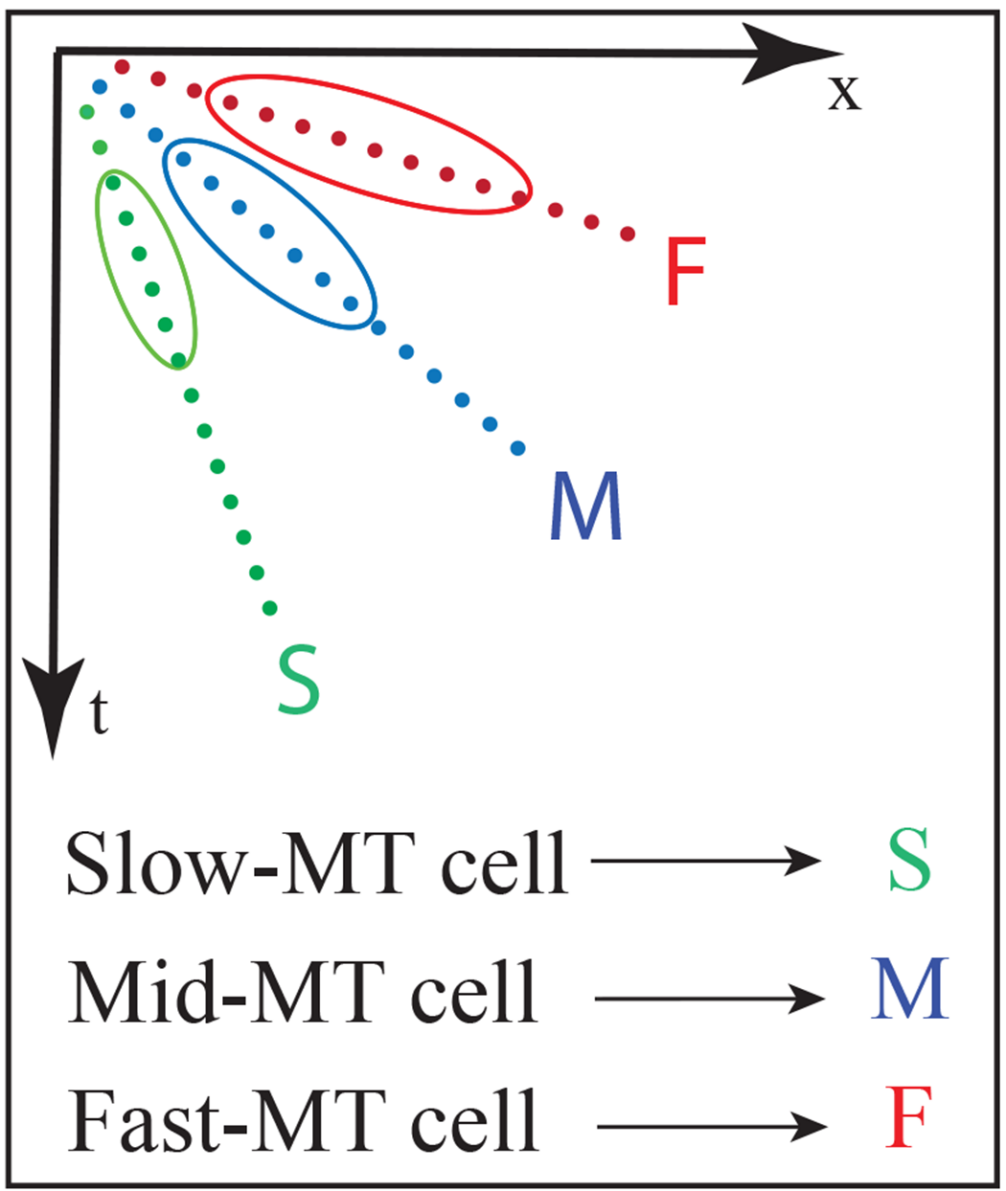
MT cell selectivity in the spatiotemporal (x, t) domain. Slow, mid and fast motion selective cells for rightward motion are depicted in green, blue and red, respectively. The diagonal lines of green, blue and red dots represent idealized event responses for slow, mid and fast input motions (with initial inputs generated from the DVS sensor). In accordance to the representation of spatio-temporal inputs increases in speed coincides with an increase in angle relative to the time axis. Model MT cells are suggested to have larger receptive field size in space in comparison to the spatio-temporally selective cells in V1. They also integrate input responses from V1 cells over a temporal period. MT cells with different speed selectivities preferentially integrate V1 responses at the proper spatial offset positions (as depicted in the elliptic outlines). The same representation occurs for leftward motions.

To verify the speed sensitivity of the MT cells, a translating stimulus with different speeds was used. The stimulus movements were recorded using DVS 128 sensor that was mounted on a tripod and placed 25 cm away from the center of the stimulus. We used a dark bar stimulus (5 × 95*mm*) moving from left to right on a light background. The bar was moved with different speeds (slow 7 *cm*/*sec*, medium 17 *cm*/*sec* and fast 23 *cm*/*sec*) using a linear actuator. Fig 5 shows the movement of the bar stimulus to the right. The events configuration that are generated by the DVS sensor is shown in Fig 5(b) in which ON-events are generated at the leading contrast edge of the bar and OFF-events at the tail edge. Fig 5(*c* _1_)–5(*c* _3_) show a translating one-dimensional signal of the bar movement in the spatiotemporal (x,t) domain in response to slow, mid and fast bar motion, respectively. The bar translation traces out a diagonal path in the spatiotemporal (x,t) domain in which the slope of the diagonal path reflects the speed of the bar movement. Cells in area V1 are driven by the input stimulus and respond in a spatiotemporal fashion. Fig 6 shows the distribution of V1 responses using box plot in which mid speed of bar movement encodes higher responses. This indicates the response of filtering stage in model area V1 is sensible to a limited speed inside a spatiotemporal frequency bandwidth which is defined by the filter weighting kernel. V1 responses are integrated in (x,y,t) space via area MT in which sizes of the RFs, or kernels, in area MT are larger than in area V1 (V1:MT 1:3, 1:3.5 and 1:4). Here, cells in area MT are modeled using elongated filters which are tuned to different speeds (slow, medium and fast motion) by oriented these filters with 300°, 340° and 355°, respectively. Hence, higher responses are generated when the RFs of MT cells match the diagonal path of motion configurations. Fig 7 demonstrates how MT cells integrate V1 responses from spatial locations along the diagonally arranged event configurations (generated by slow, mid and fast speeds of motion). The sketch outlines the initial events and the corresponding localized V1 cell responses for rightward motions occurring with different speeds. The result reveals that cells in area MT that are oriented by 300° are highly responsive to the slow motion whereas the responses of the other cells are greatly reduced with the time, see third column of Fig 5(*c* _1_)–5(*c* _3_). This is due to the fact that the two cells with higher speed selectivity (orientation 340° and 355°) are activated at the beginning and then these activations diminish over time since the RFs do not match the locations of the diagonal path of motion configurations with the cells preferred tuning. Similarly, filters that are oriented by 340° and 355° are highly responsive to the medium and fast motions respectively. The results confirm that the proposed cells respond correctly to the used speeds (slow, mid and fast).

### Motion representation in feedforward and feedback sweeps

A set of different stimuli were recorded using the DVS 128 sensor to evaluate motion representations in the feedforward and feedback sweeps. The DVS sensor is mounted on a tripod and placed 23 cm away from the center of the stimuli. These stimuli were moved via translatory and rotatory motions in which the movements of the stimuli are highlighted in the top-left of the stimuli images. To produce these motions, we used linear and rotational actuators in which the speed of the linear actuator is 20 cm/sec while the speed of the rotational actuator is 5.23 rad/sec. In order to keep a single motion hypothesis, the estimated results of the optic flow in area V1 are generated based on a weighted sum of the fundamental directions *θ*, which generates a confidence for the motion direction ${\left(u_{e}^{V1}\left(p\right)\;v_{e}^{V1}\left(p\right)\right)}^{T}=\sum_{\theta }\;r_{p,\theta }^{V1nor}\cdot {\left(cos\;\theta \;,\;\;-sin\;\theta \right)}^{T}$. The index **p** represents the spatial position **p** = (*x*, *y*). Similarly, the estimated results of the optic flow in area MT are generated based on a weighted sum of the fundamental directions *θ* and *s*_*i*_ which can be described by ${\left(u_{e}^{MT}\left(p\right)\;v_{e}^{MT}\left(p\right)\right)}^{T}=\sum_{\theta }\sum_{s_{i}}\;r_{p,\theta ,s_{i}}^{MTnor}\cdot {\left(cos\;\theta \;,\;\;-sin\;\theta \right)}^{T}$. In order to measure the accuracy of our approach, we calculated the angular error $\Phi^{V1,MT},\left(p\right)=cos^{-1}\left(v_{e}^{V1,MT}\left(p\right)\cdot v_{g}\left(p\right)\right)/\left(\right|v_{e}^{V1,MT}\left(p\right)||v_{g}\left(p\right)\left|\right)$, where ${v_{e}^{V1,MT}\left(p\right)}^{T}=\left(u_{e}^{V1,MT}\left(p\right),v_{e}^{V1,MT}\left(p\right)\right)$ and **v**_*g*_(**p**)^*T*^ = (*u*_*g*_(**p**), *v*_*g*_(**p**)) represent the estimated motion of the visual areas (V1, MT) and ground truth flow vectors, respectively. The error values in the range of [0°, 180°] are displayed as a histogram.

#### Feedforward motion representation

**Fig 8:**
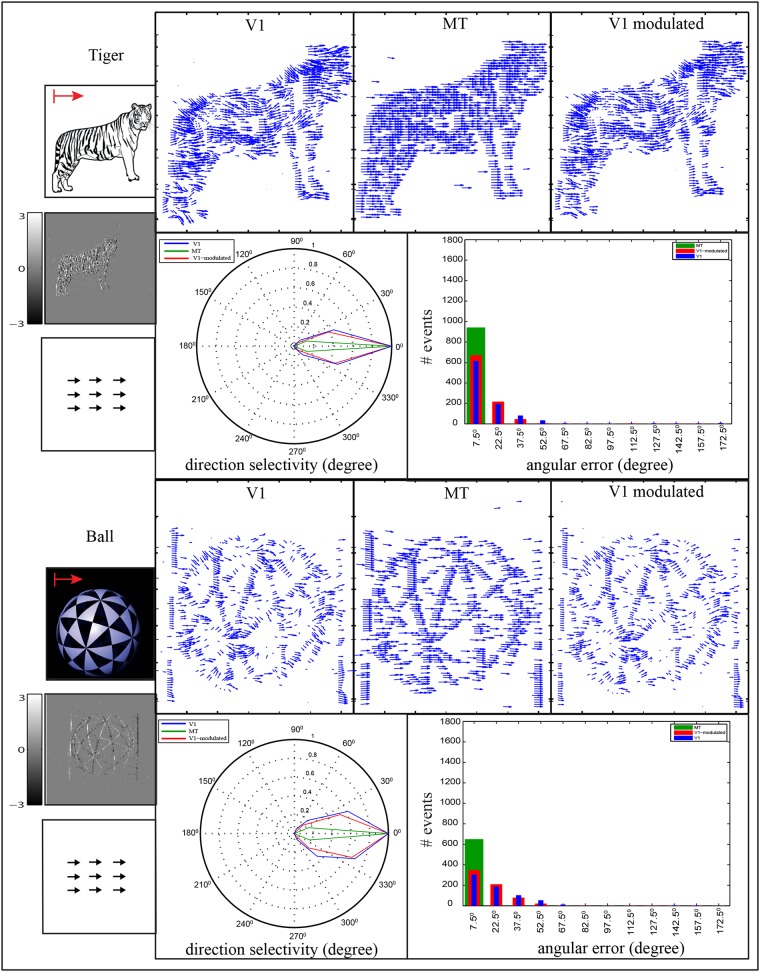
Motion estimation of tiger and ball stimuli. The stimuli are moved in rightward direction. The first column of each stimulus contains the input image, accumulated events *e* ^*on*^ and *e* ^*off*^, and a sketch of the ground truth optical flow field. The first rows of the second, third and fourth columns represent the estimated motion in areas V1, MT and the modulated V1, respectively. The direction selectivity for these areas are depicted in the polar plot where blue, green and red lines represent the responses of V1, MT and V1 modulated by MT, respectively. The histogram shows the angular error between the estimated motion and the ground truth of rightward motion direction. The abscissa of the histogram represents the binning in the range of the angular error Φ which are combined into one bar [*θ* − 7.5°, *θ* + 7.5°), and the ordinate represents the number of events. The ball image is adopted from johncarlosbaez.wordpress.com.

In the Feedforward sweep, motion is estimated via areas V1 and MT in which the normal flow that are derived from the spatio-temporal filter is weighted and integrated using the model filter mechanisms suggested for area MT. In the translatory motion, Fig 8, we used two stimuli, namely tiger and ball. The first stimulus, tiger, is characterized with a texture that comprises different striped patterns while the second stimulus, the ball, contains different slanted bars that are connected to form the ball interior. The nature of these stimuli makes the estimation complex and, then, challenging in translatory motion. The preferred direction of areas V1 and MT are depicted in polar diagrams. In tiger stimulus, the polar diagram shows that the V1 population is selective to a broad range of directions around the correct direction (0°), while this region is shrunk close to (0°) in area MT. In the ball stimulus, motion representation in model area V1 suffers from aperture problem. Here, the motion is estimated orthogonal to the bar contrast (along bar contour) while the real motion is estimated at the corner regions. Motion ambiguity (aperture problem) is reduced in model area MT due to integrate the unambiguous motion (real motion) with larger RFs. The polar diagram of the direction selectivity shows that the responses of area MT are more tuned towards (0°) than area V1. According to the error-histograms of both stimuli, the error values are reduced and concentrated at range of [0°, 15°) which indicates that motion is estimated more accurately in area MT compared with area V1. These results are consistent with physiological findings (see e.g., [39]) that showed that cells in area MT are highly directionally-selective compared to cells in area V1.

**Fig 9:**
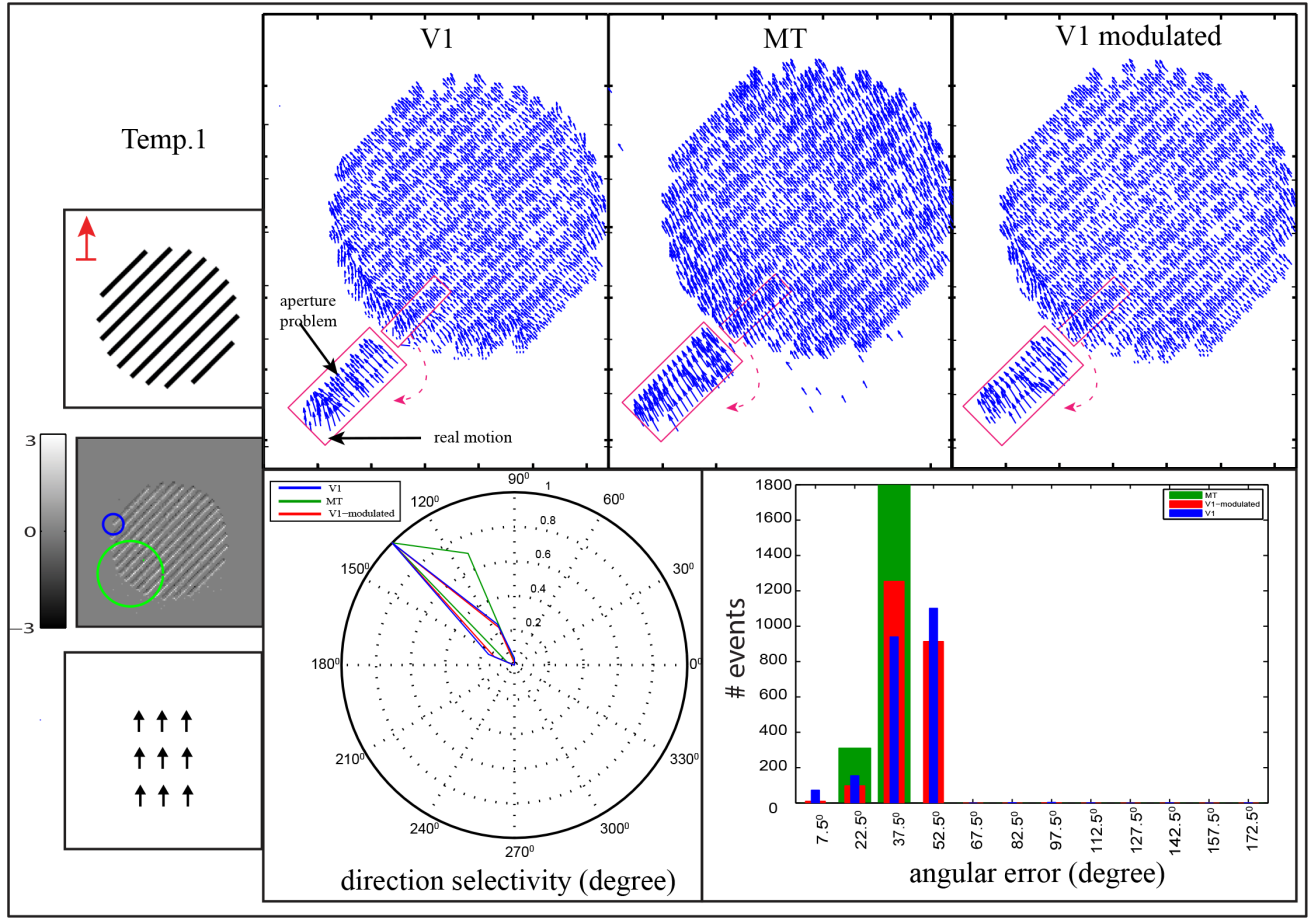
Motion estimation of bars movement through circular aperture, temp⋅1. The bars are oriented by (45°) and moved in upward direction through circular aperture. The V1 cell and one of the MT cells (V1:MT 1:3) are depicted over the accumulated events as blue circle and green circle, respectively. Motion representation in areas V1, MT and the modulated V1 for each stimulus are shown in the second, third and fourth column respectively. In this stimuli, the real motion direction is estimated at bar endings while the normal flow is estimated along bar contour. The polar plot shows the direction selectivity of V1, MT and the modulated V1 responses which are depicted by blue, green and red lines, respectively. The histogram shows the angular error between the estimated motion and the ground truth of upward motion direction. The abscissa of the histogram represents the binning in the range of the angular error Φ which are combined into one bar [*θ* − 7.5°, *θ* + 7.5°), and the ordinate represents the number of events.

Neurons in the primary visual cortex area V1 that are selective to spatio-temporal stimulus features have small RFs, or filter sizes. Consequently, they can only detect local motion components that occur within their RFs. That means along elongated contrasts only ambiguous motion information can be detected locally. It is the normal flow component that can be measured along the local contrast gradient of the luminance function (aperture problem). To probe the performance of our model to reduce motion ambiguity in area V1, we recorded a stimulus namely temp⋅1. This stimulus contains a set of black bars that are slanted with 45° and moved in a direction that differs from the normal flow as highlighted in the top-left of the stimulus. The drifting bars is seen through a circular aperture as shown in Fig 9. The result reveals that the V1 population responds in a direction (135°) which referred to the motion was estimated as orthogonal to the bar contrast. This is because the motion was estimated in a local surround, nevertheless actual motions were estimated at the bar ends. In other words, the normal flow can be computed along the local one-dimensional contour while at the ends of the bars the local two-dimensional structure enables to compute the actual motion direction. In area MT, cells integrate initial responses of model area V1 in which the sizes of the RFs are larger (V1:MT 1:3, 1:3.5 and 1:4). Such cells operate at a much larger spatial context to properly integrate localized responses. As a consequence, localized feature responses at line ends lead to stronger responses in the integration process. In all, this leads to that the preferred direction of area MT is more tuned toward the correct direction (90°). The error-histogram in Fig 9 shows that motion ambiguity is reduced in area MT in which the fourth bin [45,60) of the error values is decreased.

**Fig 10:**
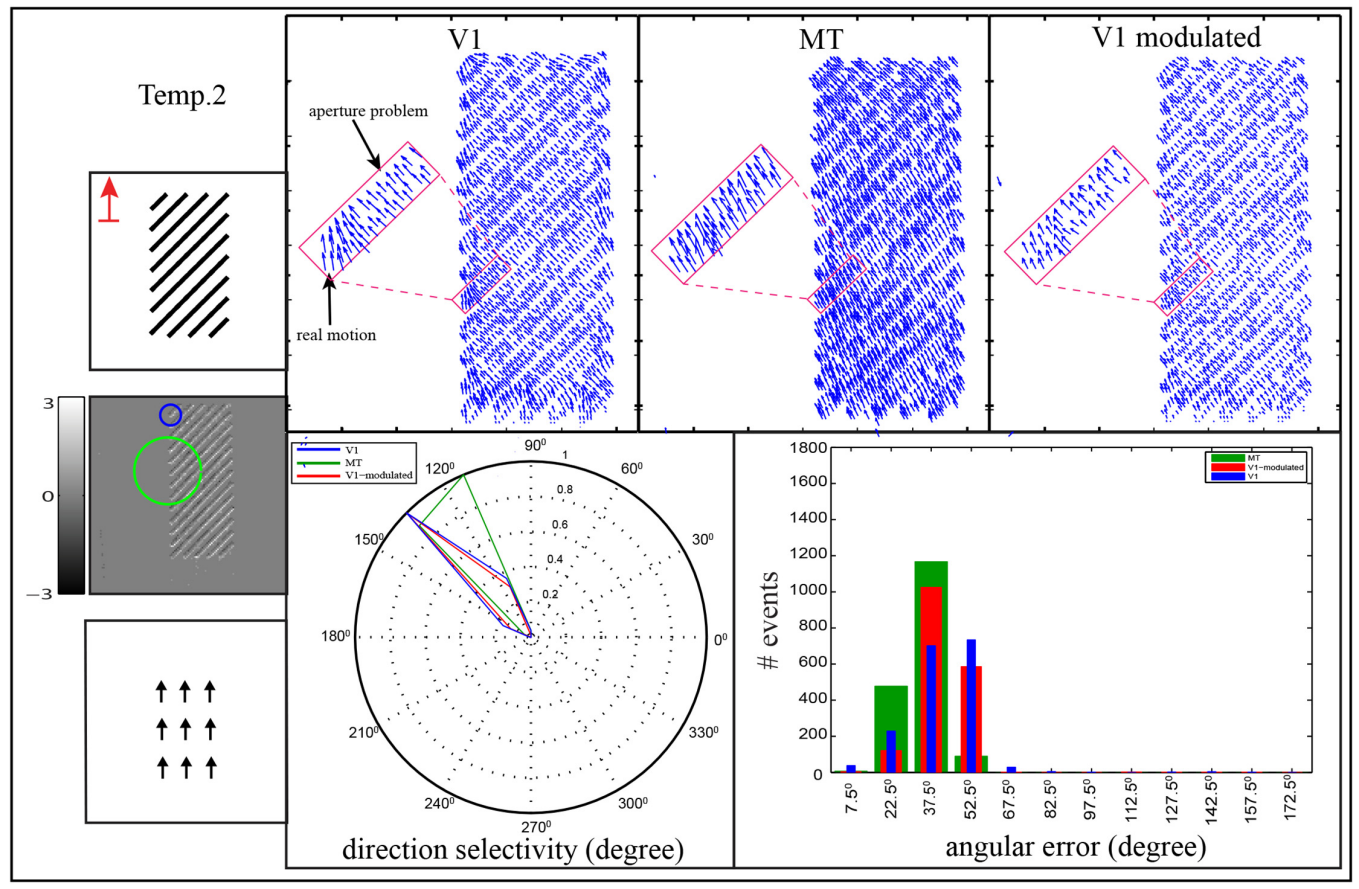
Motion estimation of bars movement through rectangular aperture, temp⋅2. The bars are oriented by (45°) and moved in upward direction through rectangular aperture with aspect ratio 5:2. The V1 cell and one of the MT cells (V1:MT 1:3) are depicted over the accumulated events as blue circle and green circle, respectively. Motion representation in areas V1, MT and the modulated V1 for each stimulus are shown in the second, third and fourth column respectively. Here, the real motion direction is estimated at bar endings which are distributed along the vertical edges of the rectangular aperture while the normal flow is estimated along bar contour. The polar plot shows the direction selectivity of V1, MT and the modulated V1 responses which are depicted by blue, green and red lines, respectively. The histogram shows the angular error between the estimated motion and the ground truth of upward motion direction. The abscissa of the histogram represents the binning in the range of the angular error Φ which are combined into one bar [*θ* − 7.5°, *θ* + 7.5°), and the ordinate represents the number of events.

**Fig 11:**
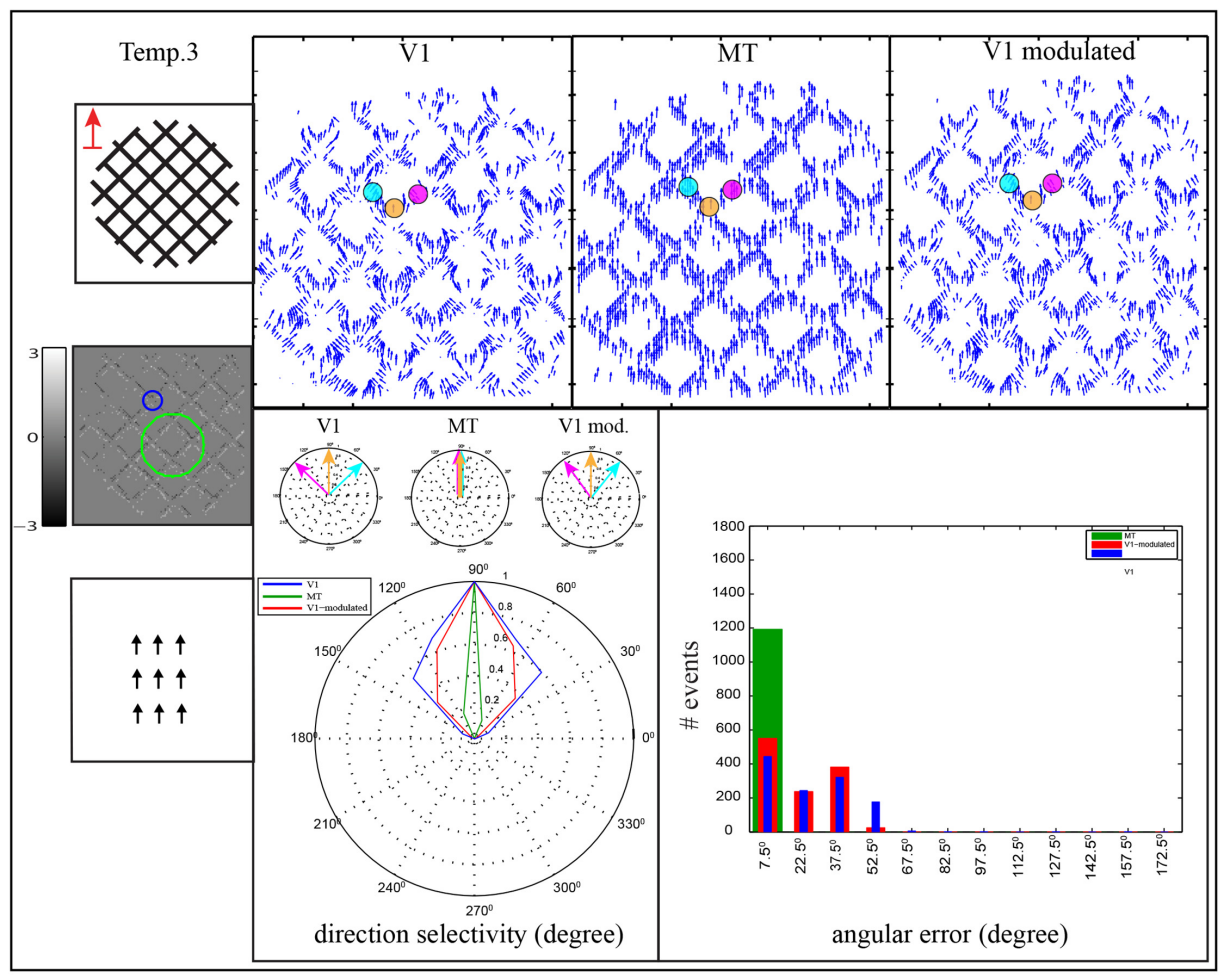
Motion estimation of 2D structure of corner terminators, temp⋅2. Two superimposed gratings are moved upward direction through circular aperture. The image input is shown in the first column of the first row. V1 cell and one of the MT cells (V1:MT 1:3) are depicted over the accumulated events as blue circle and green circle, respectively. The upper row shows the estimated motion in areas V1, MT and the modulated V1. The real motion is estimated at the 2D features (corner regions) while the normal flow is estimated along bars contours. The small polar plots show the direction selectivity of selective cells that are located on the bars contours and corner while the whole direction selectivity of the stimulus are depicted in the large polar plot. The histogram shows the angular error between the estimated motion and upward motion ground truth, where the abscissa represents the binning in the range of the angular error Φ which are combined into one bar [*θ* − 7.5°, *θ* + 7.5°), and the ordinate represents the number of events.

Object terminators, e.g. end-bar or corner, contain unambiguous motion (real motion direction), hence, the motion direction of an object can be enhanced with increasing the number of object terminators. In our test scenarios this has been investigated by recording two other stimuli, namely temp⋅2 and temp⋅3 as shown in Figs 10 and 11 respectively. In the temp⋅2 stimulus, the slanted bares (45°) are moving through rectangular aperture rather than circular aperture as shown in Fig 10. Here, the bars-ends are distributed along the vertical edges of the rectangular aperture allowing more localized real motion to be integrated in area MT. The results reveal that in area V1 the motion was estimated with preferred direction of (135°) (i.e., the motion was estimated as orthogonal to the bar contrast (normal flow)) while the preferred direction of the cells in area MT was shifted to (112°). According to our results, the responses of area MT which are driven from the rectangular aperture are more tuned toward the real motion comparing with the responses of MT cells in circular aperture. This is due to the rectangular aperture contains larger number of terminators (bar ends) that align vertically on the longer edges, which in turn leads to integrate more unambiguous information through large RFs of MT cells. In contrast, the integration process for unambiguous motion is decreased in the circular aperture due to the terminators are distributed along circular edge. The results confirm that the shape of the aperture and the number of terminators play a key rule in determining the direction of object movement. These findings are consistent with the previous studies (see e.g., [40, 41]) that showed that the direction of the perceived motion is biased towards the higher number of the terminators.

Fig 11 shows an example of 2D structure of corner terminators, temp⋅3. This stimulus contains two superimposed gratings where each grating formed by parallel lines. The stimulus was moved in upward direction which is different from the normal flow of both gratings. Here, the perceived motion is not ambiguous anymore in which the responses of both areas V1 and MT are tuned towards the real motion direction (90°). However, in area V1 ambiguous motions are estimated along bars contours (45°) and (135°) while this ambiguity is reduced in area MT. This is due to the existence of the 2D structure of the corners in which real motion can be estimated. The larger RFs of the cells in area MT allow to integrate more real motion direction that are generated at the corners regions. As a consequence, the direction selectivity of MT cells shrinks toward the real movement as shown in the polar diagram of the direction selectivity. According to the angular error histograms, values of the angular errors between the synthetic ground truth of the object movement and the estimated motion are decreased in area MT comparing with area V1.

**Fig 12:**
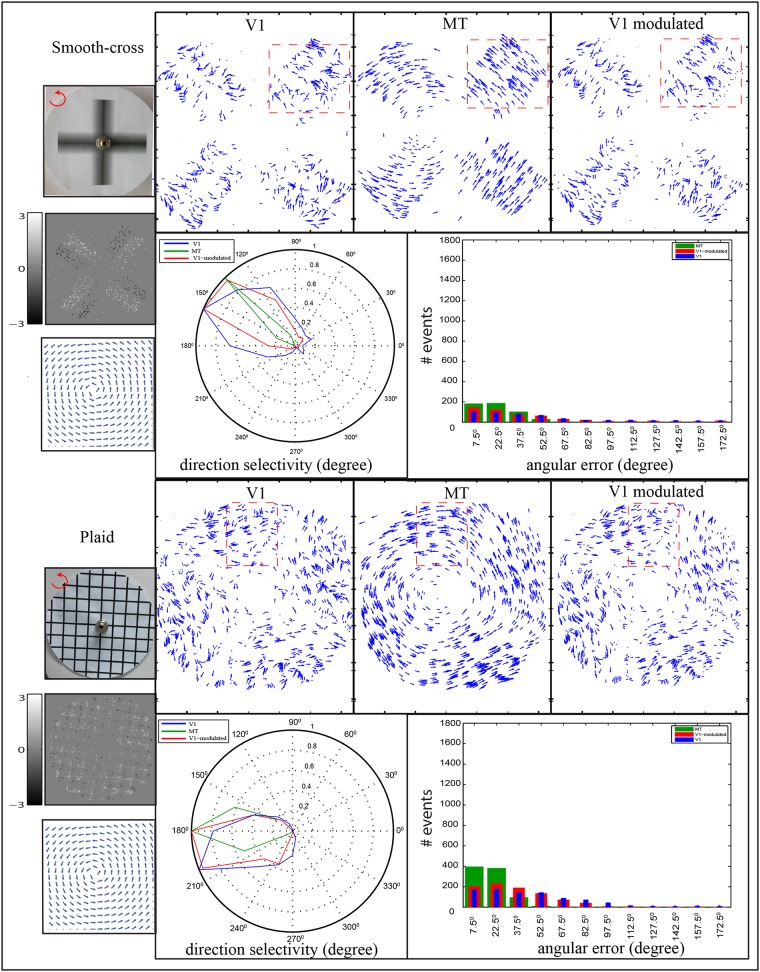
Motion estimation for smooth-cross and plaid stimuli. The stimuli are rotated in a counterclockwise direction. The first column of each stimulus contains the input image, accumulated events *e* ^*on*^ and *e* ^*off*^, and the ground truth optical flow field. The first rows of the second, third and fourth columns represent the estimated motion in areas V1, MT and the modulated V1, respectively. The polar plot shows the direction selectivity of V1, MT and the modulated V1 of the bounded region (red square). The overall errors between the estimated motion and their respective ground truth are depicted in the histograms where the abscissa represents the binning in the range of the angular error Φ which are combined into one bar [*θ* − 7.5°, *θ* + 7.5°), and the ordinate represents the number of events.

In the case of rotational motion, we used smooth-cross and plaid stimuli, Fig 12. These stimuli were rotated counter clockwise, as highlighted in the top-left of the stimulus images. The smooth-cross stimulus contains four blades with smoothed gray-level interior. These blades produce changes in intensity during the rotational motion, rendering the DVS sensor to generate ON/OFF events on the edges as well as the interior of the blades. The plaid stimulus is shaped as two sets of black perpendicular bars on white background. The generated motion components represent a challenge in terms of calculating motion direction. This is due to each set of bars generates different stream of rotational component that suffers locally from aperture problem. The input stimulus and the respective ON/OFF events are presented in the first and second rows of the first column respectively. In order to calculate the angular error for the feedforward estimated motion, we used synthetic rotational ground truth that was built based on continuous flow motion as shown in the third row of the first column. The results reveal that motion prediction via V1 area is improved in area MT in which the magnitude of the error-bins (Φ ≥ 45°) and (Φ ≥ 30°) are decreased in the smooth-cross and plaid stimuli, respectively. In the rotational sweep, the high temporal of input events delivered by the DVS sensor leads to motion components that can be considered as to mainly represent motion components tangential to a rotational sweep. However, Cells in area V1 can only measure the normal flow motion components (movement orthogonal to the bar contrast). Cells in area MT with larger receptive fields integrate these components resulting motion representation that are tangential to rotational sweep. The direction selectivity of the model areas V1 and MT are presented in the polar diagrams as shown in Fig 12. The polar diagram shows the direction selectivity in area MT is highly tuned toward the correct direction. According to the error histogram, the error values are reduced in area MT and accumulated in the error-bins of [0, 30). The reason for the high error value in rotational motion is that the rotational ground truth was built based on continuous flow motion, while our model estimates eight directions. Thus the error value can be decreased by increasing the number of estimated directions in our model.

**Fig 13:**
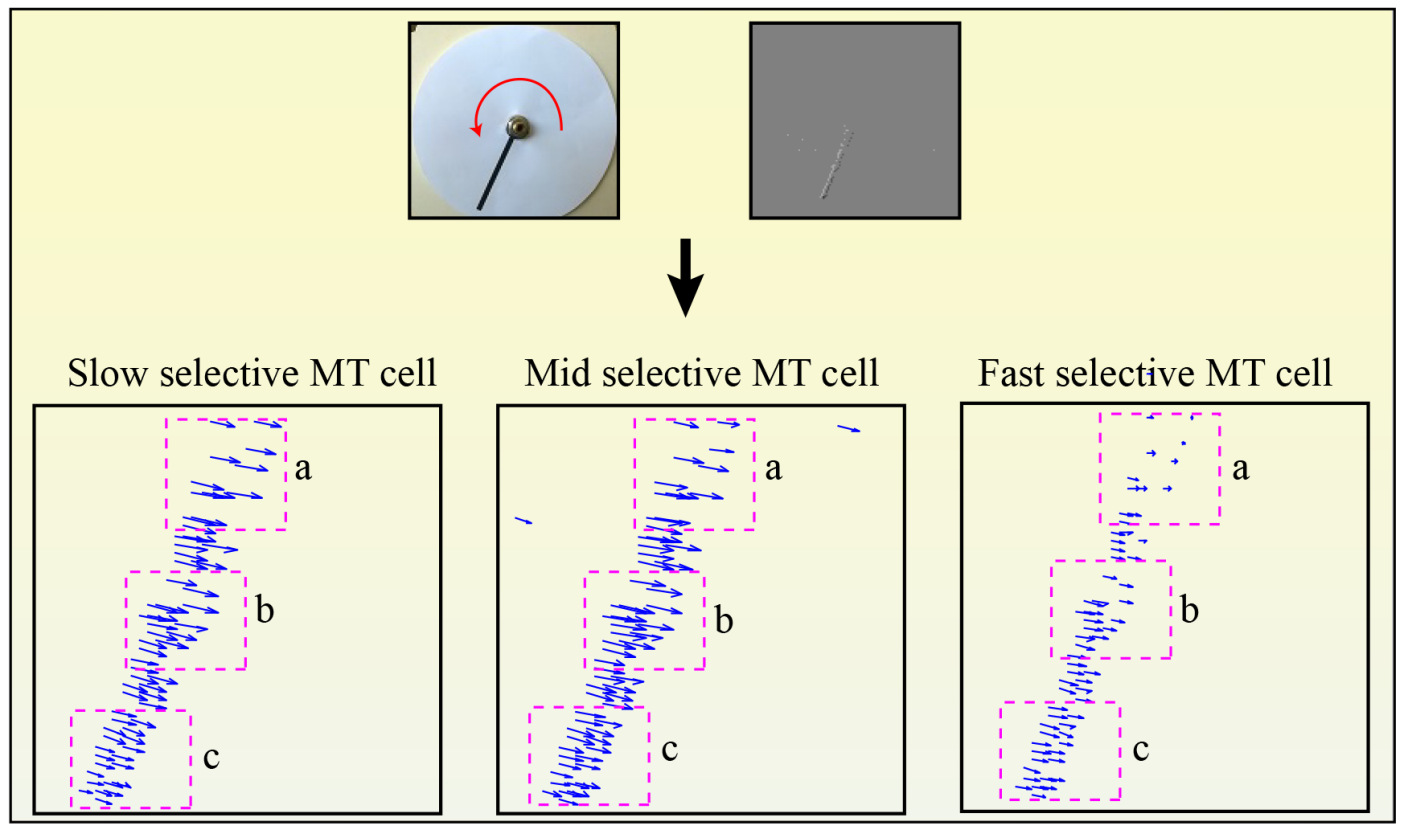
Speed selectivity of MT cells. A black bar (5 × 80 mm) is rotated counterclockwise on a white background. The first row shows the input stimulus and the ON/OFF events. The second row shows the responses of the slow, mid and fast selective cells respectively. Here, the average speeds of thee regions (a, b and c) were calculated. In the slow selective cell, the average speed at region (a) is 0.7 while the average speeds at (b) and (c) are 0.6 and 0.4, respectively. In the mid selective cell, the average speed at region (a) is 0.6 while the average speeds at (b) and (c) are 0.7 and 0.5, respectively. In the slow selective cells, the average speed at region (a) is 0.3 while the average speeds at (b) and (c) are 0.5 and 0.6, respectively.

Since the rotational motion contains different speeds as a function of the radius from the center of the motion, we utilized a rotational bar stimulus to demonstrate the speed selectivity of the MT cells, see Fig 13. Here, three regions are chosen at the bar contour in which the first region is located closed to the center (a) while the rest are located away from the center (b and c). The speeds of these points are calculated based on the length of the flow vectors $\sqrt{{\left(u_{e}\left(p\right)\right)}^{2}+{\left(v_{e}\left(p\right)\right)}^{2}}$ in which **p** represents the spatial position **p** = (*x*, *y*). The results reveal that the slow sensitive MT cell is more selective to the speed of region (a) while mid and fast sensitive cells are selective to the speed at regions (b) and (c), respectively. These results confirm that cells in area MT can differentiate different speeds of the rotational motion.

#### Feedback motion representation

In the feedback sweep, cells in area V1 receive feedback signals from the higher area MT such that the feedforward activities can be modified via the modulatory influence of feedback signals. Here, the feedback signals alone cannot excite any activation when feedforward signals are absent. In order to evaluate the impact of the MT feedback signals to enhance the initial estimation of area V1, we used the former stimuli that are used in feedforward motion representation in which the results of the modulated V1 are illustrated after 12 feedback iterations. In case of translatory motion, Fig 8, the motion estimation of the modulated V1 is shown in the fourth column. The modulated motion of area V1 in tiger stimulus shows that the re-entered signals form area MT to V1 cells enhance the initial estimation of area V1 in which the magnitude of error-bins [30°, 60°) is decreased along with increasing the magnitude of the smaller error-bins as shown in the error-histogram. In the ball stimulus, MT projection improves the initial prediction of area V1 cells in which motion ambiguity is reduced and amended to the right direction. In addition, the magnitude of the angular error-bins [30°, 60°) is decreased in the modulated responses while the magnitude of the first error-bin is increased. This confirm that the top-down feedback projection can enhance the initial motion representation of area V1 in which uncertain estimated flow is corrected.

To probe the impact of the MT feedback to reduce the ambiguity (aperture problem) of the initial estimation of area V1, we used the previous stimuli temp⋅1, temp⋅2 and temp⋅3. In the circular and rectangular apertures, Figs 9 and 10, the motion in area V1 is initially estimated orthogonal to the bar contrast (135°). This ambiguity is reduced in area MT due to cells in this area integrate the V1 activations with larger RFs. As a consequence, the feedback signal which contains disambiguate contextual information modulates the local initial responses of V1 cells. The error histograms demonstrate that the motion ambiguity in the modulated responses of area V1 is enhanced in which the magnitude of the error-bins in the range of [45,60) is decreased. On the other hand, the existence of the 2D structure of the corners in the temp⋅3, Fig 11, reduces the ambiguity of the perceived motion in which the preferred direction of cells in area MT is more tuned toward the real motion direction. Thus, the top-down signal from area MT enhances the initial local estimation of area V1 where the direction selectivity shrink toward the actual motion (90°). The error-histograms show an increasing in unambiguous motion estimation where the error of [45°, 60°) is reduced and cumulated within a small range of [0°, 15°).

In the rotational motion, Fig 12, the results of the smooth-cross and plaid stimuli show that the feedforward response from the V1 population of cells is improved by feedback modulator of area MT. The direction selectivity of the modulated V1 responses is more tuned toward the correct direction comparing with the initial estimation of area V1. The error-histogram demonstrates that the error of the modulated V1 is reduced comparing with synthetic rotational ground truth where the magnitude of the error-bins [0,30) gains more increment. The results of our model are consistent with the findings of the literature (see e.g., [34, 42]) in which the top-down modulatory signals emphasize the activities of V1 cells, which in turn, enhance the uncertain flow estimation.

## Discussion

### Summary and main contributions

We have introduced a new model for motion estimation in neural architecture utilizing the primary stages of the dorsal pathway in the primate visual cortex. We take into account event-based input that has been generated by a DVS sensor (see [43] for more details of DVS sensors). The model is inspired by the hierarchical structure of the visual system in which two cortical areas for motion perception were considered, namely the response properties of V1 and MT. These areas interact via feedforward and feedback connections in which each area builds up different feature selectivities. Areas V1 and MT were modeled using properties of direction-selective and velocity-selective cells, respectively. Here, the spatio-temporal filters are modeled to be compatible with the AER principle. The filters are inspired by the work of [16] in which the authors proposed biphasic temporal filters that can be decomposed into a convolution of a first order derivative kernel with a temporal smoothing filter. Given that the response of the DVS sensor is based on changes in the luminance of the scene, it generates an output response which related to a first order temporal derivative of the luminance input signal.

This work contributes to the state of the art in four main ways. First, by adapting the spatio-temporal filters of the type suggested by findings of [16] to be consistent with the address-events representation. In the model of Adelson-Bergen, the authors suggested to utilize temporal gamma functions of different duration in order to accomplish temporal smoothing and differentiation, leading to a temporally biphasic response shape. In order to transcribe this functionality to the spike-trains output of the DVS sensor, we make use of the following approximation: The biphasic Adelson-Bergen temporal filters can be decomposed into a convolution of numerical difference kernel (to approximate a first-order derivative operation) with a temporal smoothing filter. The event-based sensor already operates by generating discrete events based on changes, i.e. temporal derivatives, in the input signal. For that reason, we employ temporal smoothing filters which are calculated from the integral of Adelson-Bergen temporal filters (see Eq (3)) and convolve them with the input stream of events to obtain scaled versions of temporally smoothed derivatives of the input luminance function. To simplify the integral operation, we suggest to reconstruct Adelson-Bergen gamma functions by combining two temporally offset Gaussian functions. Here, the integration results of the Gaussian combinations generate new temporal filters that consistent with the spike-generation of the DVS sensor. Second, by proposing a new mechanism to simulate the functionality of the cortical area MT. The suggested neural mechanisms of area MT is motivated by principle findings of neuroscientistsâ?? studies. The localized measures from area V1 are integrated in area MT [44] through larger RFs which are roughly ten times the RFs of V1 cells [45, 46]. In our model, we integrated the responses of the spatio-temporal filters through RFs, or filters, which are larger in their size by up to an order of magnitude. However, the limited spatial resolution of the DVS sensor (128 × 128) [20] handicaps us from increasing the size of the MT filters to ten time the size of V1 filters. For that reason, we set the filters sizes with range of (V1:MT 1:≥3). This problem is however not a conceptual one of our approach and will most likely be solved with future versions of the DVS sensor. The geometry of the RFs in area MT has been investigated by [24] in which two types of RFs profiles are defined. The first type is classical receptive field (CRF) which responds best to wide field motion. While the second type is center-surround cell which is sensitive to motion contrast. In our modeling, we focused on CRF cells in which Gaussian weighting function has been used to describe the RFs profiles. This function has been used in many studies to describe the neural RF model (see e.g., [38, 42, 47]). The neural functionality of MT cells is modeled to be selective to different directions and speeds which is consistent with the physiological findings [48–50]. Here, we integrate the early motion responses of area V1 by utilizing circular RFs weighting functions with Gaussian profile. Such integration operates at a much larger spatial context to properly integrate localized responses of area V1. As a consequence, the uncertain flow estimation will be enhanced in area MT. In order to equip MT cells with different speed selectivity, we suggest to incorporate elongated RFs of Gaussian weighting function in our model. These RFs are oriented in the spatial-temporal domain which, in turn, enables the integration strategy to increase the speed selectivity of MT cells to different speeds (slow, mid and fast motions). The orientation in space-time domain with different angles encodes the speed of the input stimulation in which high oriented angles represent high-speed detectors, while small oriented angles represent slow-speed detectors. Third, by matching the properties of the feature selectivity in areas V1 and MT for feedback processing. Here, we integrated the responses over different speeds along a particular preferred direction. Such matching allows top-down feedback signals from area MT to to be compatible with the feature property of area V1. Fourth, by incorporating the response normalization in our model to achieve balance activities of individual neurons in areas V1 and MT. The interaction between the normalization of responses and the enhancement activities via feedback projection establishes the dynamics of visual cortical processing. Following the suggestion of [34] and theoretical studies (e.g., [18]) we carried out the normalization process using model neuronal activation which is described by [33] as gradual changes of the membrane potential. Activity normalization of model area V1 and MT is computed by realizing a slightly simplified version of the scheme described in [18] and solve the normalization interaction at equilibrium, namely evaluating the state response for $\frac{dv(t)}{dt}=0$. Here, the normalization process is carried out in spatial domain by calculating the pool of activities of individual neuron over a circular spatial neighborhood. In addition, we normalize neuron activities in the feature domain. Since different properties are derived from both visual areas, V1 (directions and spatio-temporal selectivity) and MT (speed and directions), we normalized responses of area V1 by averaging activities of each cell over all directions. In area MT, on the other hand, we normalized the responses by averaging activities for individual directions by integrating the activities of cells over different speeds.

As pointed out below (Section Feedforward and feedback interaction) we incorporate mechanisms of modulating feedback which enhances driving feedforward activations. In a nutshell, the architecture enhances signals which match along the feedforward and the feedback pathways while those feedforward signals that do not cohere with top-down predictions are reduced. In order to augment the modulatory feedback mechanisms with a mechanism to reduce and even extinguish activations, a context sensitive down-modulating mechanism needs to be employed. The proposed normalization stage that operates upon a pool of cells in the surrounding neighborhood of spatial and feature selectivity serves a mechanism to reduce the overall activation. The normalization tends to conserve the overall signal energy in the pool of cells which competitively interact. The prior enhancement of selected cells in turn reduces the activity of those cells that have not received any feedback.

### Feedforward and feedback interaction

The feedforward and feedback hierarchical model in this work considers two main areas of the visual system, V1 and MT. The structural model of each of such visual areas is defined by three stages: (i) a stage of initial input filtering, (ii) a stage of activity modulation of the filtering responses via top-down feedback signals, and (iii) a stage of activity normalization in the spatial and feature domains to achieve balanced activations of a target cell against a pool of neighboring cells. The structural model proposed here can be transcribed to the cortical areas architecture that are suggested in [51]. Each of such areas has a specific filtering stage model to generate the driving feedforward signal with particular features. Feedforward signals propagated from area V1 drives the direction feature while the feedforward signal of area MT drives the velocity feature (direction and speed). This framework of feature selectivity of areas V1 and MT is consistent with experimental findings [6, 50, 52]. The activations of each area are normalized using a divisive mechanism suggested in [53–55]. Here, the same principle is utilized in which the normalization operation uses contextual information from a local neighborhood that is defined in space as well as feature domain.

Higher-level areas in the visual cortex send feedback signals that are re-entered to the earlier areas in the visual hierarchy [56, 57]. Different hypotheses for cortical feedback have been discussed in the literature in which two major hypotheses have received different support from the experimental evidence [58]. These hypotheses are defined as modulatory (biased competition) and predictive coding feedback. In a nutshell, modulatory feedback suggests that signals in the feedforward stream are enhanced by feedback projection. This feedback projection driven gain control mechanism to bias subsequent competition between neurons which leads to enhance responses patterns [59–62]. While predictive coding aims to reduce the residual error between the feedforward signals and feedback projection in order to approach sensory prediction that generated via higher level of processing [63–65]. Further evidence shows that feedback processing tends to act as a modulator that amplifies the neuronal spiking signals at the level of cortical pyramidal cells [10]. In our model, we adopted the concept of modulatory feedback (biased competition). Here, the responses from lower level cortical area V1 are modulated via feedback projection of the higher area MT. Feedback modulation process is thereby carried out in such a way that feedback signals could not provoke any activity in the absence of the feedforward signals.

Although, the neuroscientists’ studies confirm the impact of feedback processing stream among cortical visual areas, the precise function of feedback role is still not fully uncovered. A fundamental question here is how MT cell responses project activations along their feedback stream to area V1, since cells in area V1 are selective for direction while cells in area MT are selective for both speed and direction? This question directly addresses the suggestion made by [57] that maps in different (cortical) area re-enter their signal. We proposed integrating the responses over different speeds along a particular preferred direction. As far as we know, this is the first event-based mechanism to address feedforward and feedback interactions between two areas that have different feature selectivity. In addition, response normalization for areas V1 and MT was embedded to obtain balanced activities in both areas. Here, we implemented the normalization at equilibrium state. The activities of the cells were normalized in the spatial domain through a distance weighting dependency filter (fall-off function), where the size of the filter is larger than the size of the cell RFs. In addition, we regulated the responses over feature space by calculating the average activity over all directions.

### Relation to previous models of motion estimation

Motion estimation is an interesting topic that is investigated intensively using conventional frame-based cameras (see, e.g., [16, 66–70]). Relatively few studies have been reported how to transcribe the functionality of such classical approaches to be consistent with neuromorphic vision sensors (see [20] for more details about neuromorphic vision sensors). In [71] the authors used a least squares error minimization technique introduced in [66] to estimate the motion using an DVS sensor. Due to DVS sensor generates a stream of events (ON or OFF) and does not provide gray levels, thus, the authors have been suggested to use pixel activities by integrating events within a short temporal window. Benosman and co-authors showed beneficial results for motion estimation, however, their numerical approximation of the local gradients of the luminance function from event-sequences has its limitations and may lead to inconclusive results (see [23, 47]).

In [72], the authors introduced an algorithm for motion estimation using a DVS sensor in which spatiotemporal filters of the type suggested by findings of [73] were utilized to estimate a local motion for each generating event in the scene. The authors implemented the spatiotemporal filters using a spatial buffer in which the timestamp of each event is stored. Recently, Tschechne and co-authors extend their work in [47] by introducing a framework of a hierarchical architecture of multi-stage motion detection and integration in which V1 responses are integrated in area MT over a larger neighborhood using circular RFs. Here, the authors have focused on motion direction selectivity in area MT. The implications of event-based sensing in the context of visual motion have been investigated by [23]. Brosch and co-authors discussed different principal approaches for optical flow detection. They showed that gradient-based methods for local motion detection in principle suffer from the sparse encoding in address-event representations (AER) because they are rare with respect to a local weighted integration during filtering. The authors further investigated approaches to exploit the local plane-like structure of the event cloud and how local filtering can be properly defined.

The weighted intersection mechanism (WIM) sensor has been proposed by [74] and developed in [3]. Here, a motion sensor built up in stages from two spatiotemporal filters with properties based on V1 neurons. The sensor mechanism incorporates two V1-like units based on spatio-temporal energy filters. The first unit has sustained low-pass temporal frequency tuning (referred to non-directional type), whereas the second unit has transient band-pass temporal tuning (referred to directional type). This mechanism enables two filters with broad temporal tuning (one low-pass and the other band-pass) to be converted into a filter with tight temporal frequency tuning and an orientation that maps onto the oriented spectra generated by moving edges. Perrone and co-authors showed that the speed tuning property of such a WIM filter is comparable to that found in many MT neurons.

Feedback processing tends to act as a modulator input from higher areas that mediate top-down contextual effects [8]. Such processing has been demonstrated to enhance the gain of neural representations during different processing phase in several tasks such as texture segregation (figure-ground segregation) [75] as well as increasing the visual awareness [76]. The feedback modulation hypothesis, No-Strong-Loops, implements a distinct driving and modulating inputs principle (see [77]). The driving input can strongly activate the neurons that are concerned with sensory input processing, or filtering. The modulatory inputs, on the other hand, cannot generate any activity by themselves but can modify the driving input of a target neuron utilizing contextual information provided by higher-level representation. The modulatory feedback is considered as a common principle that has been observed in many studies. In [34, 42] a model of motion processing in areas V1 and MT has been proposed in which driving feedforward and modulating feedback signals interaction are considered for the purpose of motion detection and integration. In the model, the localized motion response in area V1 was integrated in area MT via feedforward processing stream while in feedback processing the initial estimation of area V1 was modified via feedback projection from area MT. The authors showed that top-down modulatory signals emphasize the activities of V1 cells, which in turn, enhance the uncertain flow estimation and improve the visual motion segregation. In [78], the authors presented a hierarchical architecture of cortical feedforward and feedback computation where they proposed how a top-down feedback-modulatory learning mechanism can increase the gain of the feedforward driving inputs. This results in network feature representations that automatically adjust the connection weights utilizing unsupervised learning. As a result the top-down predictions are improved for the input pattern.

The motion boundary contour system (or Motion BCS) model have been introduced in [79] and [80]. The authors suggested a neural model for the purpose of motion detection and integration. The input of the model taken to be the outputs of FACADE mechanisms (Form And Color and DEpth processing) which is firstly described by [81]. This model has been extended by [82] where several processing stages with a feedback interaction between MT and MST stages are presented. Grossberg and co-authors showed how the aperture problem can be solved based on 2D feature signals. This has been accomplished by computed unambiguous motion from feature tracking points which are amplified before they propagate across position and are integrated with ambiguous motion signals within bar interiors. Grossberg and co-authors demonstrated how the feedback signals from model MST to MT cells which encode the winning direction boost directionally consistent cell activities and suppress inconsistent activities over the spatial region to which they project. A model of recurrent motion processing between areas V1 and MT has been suggested by [42], where the authors suggested a model of V1-MT feedforward and feedback processing in which the model of each area consists of three steps, namely feedback modulation, feedforward integration, and lateral inhibition. The initial motion detectors in area V1 consists of a set modified elaborated Reichardt detector. In feedforward processing the localized motion representation in area V1 were integrated via model area MT using larger receptive fields (V1:MT, 1:5). In the feedback processing the initial estimation of area V1 were modified via the feedback projection from area MT. The authors showed how ambiguities of detected visual motion can be solved by combining mechanisms of local lateral interaction with modulatory feedback.

In [34], a model of motion processing in areas V1 and MT has been proposed in which driving feedforward and modulating feedback signals interaction are considered. Here, the localized motion response in area V1 were integrated in area MT via feedforward processing signals while in the feedback processing signals the initial estimation of area V1 were modified via the feedback projection from area MT. The authors showed that top-down modulatory signals emphasize the activities of V1 cells, which in turn, enhance the uncertain flow estimation and improve the visual motion segregation.

Our model differs from these other approaches in the initial method of motion detection. Here, we adopted the bio-inspired model suggested in [16] and adapted the filtering principle to make the approach consistent with the functionality of DVS sensors. The event-based technology of visual sensors provides our model with a high temporal resolution (1 *μs*). In addition, the redundant information captured by conventional frame-based camera is reduced. The architecture of the proposed model takes into accounts feedforward and feedback interactions in which area V1 are modeled as direction selective cells while area MT modeled as velocity (speed and direction) selective cells. Here, V1 responses are integrated via elongated RFs that are tilted in space-time domain. This kind of integration increases the cells sensitivity to detect different speeds, fast, mid and slow. Our model take into accounts feedback processing and focuses on how context information from higher-level area can re-enter into lower-level area in which both areas have different feature selectivity. This has been done by integrating the responses of MT cells over different speeds to match the properties of both areas, V1 and MT.

### Model evaluation and future work

To verify the speed sensitivity of the proposed elongated RFs, a translating bar with different speeds (slow 7 *cm*/*sec*, medium 17 *cm*/*sec* and fast 23 *cm*/*sec*) was used. The results demonstrate the correct speed sensitivity of the suggested cells. Our model was tested using different kinds of stimuli moving in different directions. In feedforward processing, the results show that V1 population responses are selective to broad directions. These responses are enhanced in area MT in which the responses are highly tuned to the correct directions. This outcome is consistent with the functionality of MT cells which increases the direction selectivity (see e.g., [39]). Feedback modulation processing, on the other hand, improves the direction selectivity of the initial motion estimation via area V1. However, the aperture problem has not been completely solved. Further work is needed to resolve this issue.

We demonstrated the angular error for the bottom-up and top-down predictions using a histogram, where the error was calculated by comparing the estimated flow with synthetic ground truth. The stimuli results show an improvement in the flow estimation in area MT and modulated V1, compared with the initial motion estimation in area V1.

Our model can be extend by adding other functions of the cortical areas such as medial superior temporal (MST). This will bolster the model’s ability to process more complex motions in which the cells in this area have larger RFs than area MT and can respond to complex patterns of visual motions. In addition, the projection feedback from this area to MT will enhance the response activities of MT cells. The architecture of our model can be used as a basic adaptive scheme for motion estimation based on sparse event-based input. It is thus conceivable that other researchers interested in biologically inspired technology based on address event representation may start from this point in order to further develop mechanisms in this framework.
